# Availability, access and feeding practices associated with malnutrition in children under 24 months in Mozambique*

**DOI:** 10.1590/1518-8345.7366.4602

**Published:** 2025-11-03

**Authors:** Florência Custódio Mondlane, Lara Morena Cardeal, Rogério António de Oliveira, Maria Rita Marques de Oliveira

**Affiliations:** 1 Universidade Estadual Paulista, Faculdade de Medicina, Botucatu, SP, Brazil.; 2 Scholarship holder at the Coordenação de Aperfeiçoamento de Pessoal de Nível Superior (CAPES), Brazil.; 3 Universidade Estadual Paulista, Instituto de Biociências, Botucatu, SP, Brazil.; 4 Scholarship holder at the Conselho Nacional de Desenvolvimento Científico e Tecnológico (CNPq), Brazil.

**Keywords:** Child Nutrition, Malnutrition, Breast Feeding, Food Security, Food Production, Eating Practices

## Abstract

to analyze the association between malnutrition and the availability, access and feeding practices of children under 24 months in Mouha, Mozambique.

observational, cross-sectional, analytical study with a sample of 284 children under 24 months of age whose heads of household and mothers or those responsible for feeding the child answered structured forms at home. Multinomial log-linear models were used to verify the association of predictor variables with anthropometric indicators of malnutrition.

it was observed that less than one third of the children were free from malnutrition. Only 23% of the children had adequate nutrition that met all the criteria for their age group, which was negatively associated with severe acute and chronic malnutrition; 67% of the children were exclusively breastfed, and the prevalence of continued breastfeeding occurred between 88% and 95%. The existence of food reserves was a protective factor against chronic malnutrition at all degrees, confirmed by the practice of agriculture and the diversity of animals raised. The feeding problem was associated with acute and chronic malnutrition in children over one year old.

malnutrition in children under 24 months old is associated with dietary adequacy, availability of food for consumption and access to it.

## Highlights

**(1)** Households are predominantly made up of farmers. **(2)** Malnutrition is the result of inadequate feeding practices. **(3)** Children over one year old were less protected from malnutrition. **(4)** Less than a third of children were free from malnutrition.

## Introduction

The human right to adequate food is included in the main international agendas and represents one of the most challenging objectives of sustainable development, interdependent with all others. Hunger, malnutrition and other forms of malnutrition have affected more than half of the world’s population. In 2022, hunger affected between 691 and 783 million people worldwide, according to United Nations estimates. Although some progress has been made in other affected regions, hunger continued to increase in all regions of Africa, mainly affecting rural residents and households headed by women^(1)^.

Across the world, more than 22.3% of children were stunted in 2022, with 6.8% being underweight and 5.6% being overweight. In Mozambique, there were 2 million children with stunted growth, which represented approximately 5.3% of its total population^(1)^. It has been estimated that the prevalence of chronic malnutrition in preschool children in the country was 37%^(2)^. This situation has been aggravated by inadequate access to water and sanitation, especially in the northern region of the country^(2)^. Good nutrition in the first years of life is essential for the full development and growth of children, with a view to ensuring a full and healthy life until adulthood^(3)^.

Anthropometric measurements and their derived indices are used as indicators of nutritional status, are easy to obtain and sensitive to changes in body composition, and are widely used to assess and monitor the nutritional health of the population^(4-5)^. Recent protein-calorie insufficiency is associated with thinness and low weight, and emaciation can mean acute malnutrition. On the other hand, a constant lack of these nutrients, for long periods, results in impaired child development, indicated by short stature or chronic malnutrition^(6-7)^.

Maternal and child malnutrition has short- and long-term consequences. Morbidity and mortality and impaired physical and psychosocial development are short-term consequences, resulting in increased infant mortality and susceptibility to disease. In the long term, malnutrition can result in cognitive difficulties, worsening reproductive performance, incidence of overweight and metabolic diseases in adulthood as a consequence of hunger in childhood^(8)^.

Malnutrition has been assessed based on immediate, underlying and basic causes: inadequate food intake and diseases are among the immediate causes of malnutrition, while food insecurity and inadequate health care and attention are among the underlying causes. The basic causes include lack of access to public policies (health, education, work, land) and technologies, conditioned by insufficient financial and human capital and the physical and social conditions of the context in which one lives^(9)^.

Food and Nutrition Security (FNS) is a preponderant factor in ensuring good nutritional status. FNS exists when all people, at all times, have physical, social and economic access to safe, nutritious and sufficient food that meets their dietary needs and food preferences for an active and healthy life, based on health-promoting eating practices, respecting cultural diversity, that is, environmentally, culturally, economically and socially sustainable^(10)^. This concept involves the dimension of food availability in the environmental context; physical and financial access in the family context; use in the biological, affective and cultural context; stability and sustainability of the first three and the subject’s right to action^(10)^.

Studies have shown that family farming for self-consumption contributes positively to the health and livelihood of families and their children and to the reduction of food insecurity^(11)^. However, given the complexity of the process of social determination of malnutrition, there is a need to deepen its understanding, taking into account cultural, social and environmental issues, whether related to means of subsistence or dietary practices^(12)^.

In the case of children, the role of the mother or caregiver is very relevant. It was observed, for example, that exclusive breastfeeding among children aged 0 to 5 months and adequate nutritional transition were more impaired in children whose mothers worked outside the home^(13)^. But that is not all, it is also worth noting that, in Mozambique, diets are mostly based on cereals, which has limited the dietary diversity of children aged 6 to 24 months^(14)^.

Mouha is a town in the Sussundenga district, in the Manica province of Mozambique, a region that has been severely affected by climate events, but where the most food is produced in the country, however, it is also one of the regions most affected by child malnutrition^(15)^. It has been postulated that child malnutrition in the region may be due to inadequate feeding practices, usually from mothers or those responsible for feeding the child. In the literature related to Portuguese-speaking African countries, the existing information on malnutrition corresponds to data from cross-sectional studies that do not delve into the causes of malnutrition^(16)^. It is not yet known whether children affected by malnutrition are subject to low availability of food and little access to it or whether feeding practices are inadequate. Given the complexity of the process of determining malnutrition and the lack of data from the region, this study started from the hypothesis that variables related to the determination of nutritional status regarding availability, access and consumption could be producing child malnutrition in Mouha, as probably in other locations with similar characteristics. Thus, the aim of this research was to analyze the association of malnutrition with the availability, access and feeding practices of children under 24 months old in Mouha, Mozambique.

## Method

### Study design

This is an observational, cross-sectional, analytical study that involved collecting information through a household survey. The STROBE (Strengthening the Reporting of Observational Studies in Epidemiology) checklist was followed to write this document.

### Study population and setting

The population consisted of children under 24 months old, the head of the household and the mother or person responsible for feeding the child, living in the town of Mouha, Sussundenga district, Manica province in Mozambique, whether or not they practiced agriculture and livestock farming. The economy of the district is based on agriculture, and the town is made up of 14 neighborhoods, a type II health unit (a small health unit that provides primary health care in rural areas) and four primary schools.

### Selection criteria

The heads of household, mothers or those responsible for feeding children under 24 months of age registered at the health unit (HU) were invited, regardless of the length of residence in the location, who did not have health problems that directly affected their nutritional status (HIV/AIDS, tuberculosis, delayed psychomotor development), as indicated on the child’s health card or prenatal form. All Family Groups (FG) (this is a specific designation, with its own legislation, for a family grouping in Mozambique, not common in Brazil) who did not complete the survey data and/or children diagnosed, a posteriori, with problems that directly affected their nutritional status were excluded from the study.

### Sample and sampling

A simple random cluster sampling plan was used, with a power of 80% and a significance level of 5%, considering 22 independent variables to be analyzed by multiple linear regression^(17)^, which resulted in 234 children in their respective households, with only one child from each household. A 20% loss rate was considered (n=340), and in the end, 284 households from 10 of the 14 existing neighborhoods were included in the research. First, the leaders and/or secretaries were contacted, subsequently and with due authorization, we invited families with children under 24 months to participate in the research.

### Instruments used and study variables

Two structured forms were used to collect data, one applied to the head of the household or his/her representative and the other to the mother or person responsible for feeding the child. The form addressed to the head of the household consisted of the following sections: A - with questions related to the demographic data of the head of the household; section B - with questions related to crop and livestock production; section C - with questions about livelihoods; section D - with questions about food consumption, including the 24-hour recall and the one-week recall; section E - with questions about food insecurity assessment, in which the FIES - Food Insecurity Experience Scale, developed by the Food and Agriculture Organization (FAO) as a global tool, including validation for Mozambique^(18)^, was applied. The second form was addressed to the mother or person responsible for feeding the child, and consisted of sections A and B; in section A, the questions were about the children’s demographic and anthropometric data, and in section B, questions about exclusive breastfeeding, continued breastfeeding, and complementary feeding in the form of a 24-hour and one-week recall of the food groups consumed.

Both forms were obtained from the data collection instrument of the Technical Secretariat for Food and Nutrition Security (version 02/21/2018)^(19)^, consisting of predefined answers, such as: yes, no, don’t know, short answers, and multiple-choice answers corresponding to the participant’s reality.

The foods were grouped according to the FAO food classification, as follows: cereals; vegetables and tubers rich in vitamin A; dark green vegetables and leaves; other vegetables; legumes; orange or yellow fruits; other fruits; meats (white and red); eggs; milk and dairy products; fish and seafood; offal; seeds; oils and fats; sweets and sugar; seasonings and condiments^(20)^.

### Study variables

The study variables were organized into four dimensions:

Demographic variables: 1) Age of the household head, 2) Household head sex 3) Household head educational background, 4) Number of members in the household, 5) Sex of the child, 6) Number of children under 24 months old in each household and 7) Age of the child.

Nutritional status: 8) Weight/Age, 9) Weight/Height, 10) Height/Age and 11) Presence of bilateral edema (no cases found).

Food availability: 12) Agriculture practices, or not, 13) Variety of crops, considering greens (cabbage, lettuce, amaranth, pumpkin leaves, other); vegetables (carrot, tomato, pepper, okra, cabbage, other); legumes (beans, peanuts, other); cereals (corn, sorghum, wheat, rice, other); oilseeds (sunflower, sesame, coconut, other). 14) Crops for sale: greens (cabbage, lettuce, amaranth, pumpkin leaves, other); vegetables (carrots, tomatoes, peppers, okra, cabbage, other); legumes (beans, peanuts, other); cereals (corn, sorghum, wheat, rice, other); oilseeds (sunflower, sesame, coconut, other), 15) Duration of the reserve, 16) Cultivation difficulties, 17) Variety of animals, 18) Animals for sale, 19) Food adequacy.

Food access: 20) Main source of income: production, sale of crops and animals; self-employment (processing, salary, pensions, remittances, trade, services) and food assistance/help/earnings/odd jobs, 21) Feeding difficulties, number of months with feeding difficulties, 22) Food insecurity, feeding practices, 23) Compliance with national recommendations, 24) Exclusive breastfeeding, 25) Continued breastfeeding, 26) Introduction of solid, semi-solid or soft foods, 27) Minimum dietary diversity and 28) Minimum frequency of meals. It is important to clarify that the child considered as having adequate nutrition was considered as “yes” if, according to their age, they receive adequate nutrition in accordance with the recommendations of the World Health Organization (WHO), the United Nations Children’s Fund (UNICEF) and the FAO (adopted by the Ministry of Health of Mozambique), with exclusive breastfeeding from 0 to 5 months, continued breastfeeding from 6 to 24 months, minimum dietary diversity and a minimum number of meals, according to their age.

To analyze food insecurity, the questionnaire and Excel® analysis template provided by the FAO^(21)^ were used, which include instruments validated for Mozambican Portuguese and in two dialects. The demographic variables, nutritional status, food availability and food access were considered as independent variables, and the outcome variables were the nutritional indices. For analysis purposes, the following were considered: age of the household head; household head educational background; number of members in the household; age of the child; the number of children under 24 months in the HH, and the total number of months with feeding difficulties were stratified by categories.

### Data collection and period

Data collection was preceded by a pilot study to assess perceptions of the issues and interpretation of the data, and by a brief presentation by the researcher to the local head and neighborhood heads. Data collection began on January 16th, 2023, from the neighborhood closest to the local headquarters to the furthest away and from the furthest to the closest household, with the exception of one of the neighborhoods closest to the headquarters, which was one of the last to be visited due to a lack of means of communication with the local leadership. First, the neighborhood leader informed the families about the research that would be carried out and the dates scheduled for data collection in each neighborhood, and then the researcher went to the home of each household, accompanied or not by the neighborhood leader. The average length of the interview with each head or representative of the households was 20 minutes, and 40 minutes with the mothers or those responsible for feeding the children, including the anthropometric assessment.

### Ethical aspects

The research began after approval by the National Bioethics Committee for Health of the Ministry of Health of Mozambique, in Ref. 882/CNBS/22, and after obtaining the Free and Informed Consent Form (FICF) for older participants and consent from mothers or those responsible for feeding children under 18 years old. All information after being transcribed, analyzed and interpreted was destroyed, and it should be noted that the data were anonymized for analysis.

### Data analysis

The collected data were organized in a spreadsheet. Subsequently, in order to identify patterns and understand the nature of the data, descriptive analyses were performed using the statistical programming language R, *dplyr* package^(22)^. The proportions obtained as results for the qualitative and quantitative variables were organized in tables, and the demographic, food availability (except for the variable duration of the reserve, which was expressed as a mean) and food access variables were categorized and expressed as a percentage.

In order to investigate the association between the outcome and predictor variables, the data were divided for the analyses. The first analysis was performed by adjusting multinomial log-linear models, considering the data of all children in months. Similarly, the second adjustment of the multinomial log-linear model was performed, considering the data that had a child’s age category with the filter of 12 to 24 months. In this context, we considered each nutritional index as an outcome variable, with the aim of determining which predictor variables had the greatest impact on the outcome variables, considering a 5% significance level.

## Results

Out of the 284 studied families, most heads of households were ≥ 41 years old (34%). Of the total, 88% were male, 51% had seven years of schooling and 4% had not attended school; more than 50% of households were composed of six or more members and, in the majority (96%), there was only one child under 24 months old. Of the total of children, 51% were male and the majority (44%) were between 6 and 24 months old (Table 1).

**Table 1-:** Household demographics (n = 284). Mouha, MN, Mozambique, 2023

**Variables**	**Categories**	**Total**	**Percentage**
HH* head age in years (in years)	≤ 20	3	1%
	21-25	55	19%
	26-30	55	19%
	31-35	41	14%
	36-40	34	12%
	≥ 41	96	35%
HH* head sex	Male	250	88%
	Female	34	12%
HH* head educational background (in years)	None	12	4%
	Up to 7 years of study	127	45%
	More than 7 years of study	145	51%
Number of people in the HH*	≤ 5	132	47%
	6 to 10	130	46%
	≥ 11	22	7%
Children’s sex	Male	144	51%
	Female	140	49%
HH* number of children who were less than 24 months old	1	273	96%
	≥ 2	11	4%
Children’s age (in months)	0 to 5	80	29%
	6 to 8	41	14%
	9 to 11	38	13%
	12 to 24	125	44%

*HH = Household

Out of the 284 children evaluated, 51% were classified as having adequate length for their age, 60% as having adequate weight for their length and 63% as having adequate weight for their age. However, when considering the set of indicators with children distributed by age group, the proportion of children with all adequate index was lower, ranging from 42% among those aged 6 to 8 months old to 21% among those aged 12 to 24 months old, as shown in Table 2.

Regarding feeding practices, when analyzing adequacy according to the guidelines adopted in the country for each age group, only 66 (23%) of the children met all the criteria. Among children aged 0 to 5 months, 67% were exclusively breastfed, among those aged 6 to 8 months, 88% were on continuous breastfeeding, 56% had a minimum meal frequency and 15% had a minimum dietary diversity. In the age group of 9 to 11 months, 95% were on continuous breastfeeding, 30% and 45% had a minimum dietary diversity and minimum meal frequency, respectively. Of the 125 children aged 12 to 24 months, 88% were on continuous breastfeeding, 24% had a minimum dietary diversity and 59% had a minimum meal frequency (Table 2). The survey of food availability in the households showed that most families practiced agriculture, with cereals and vegetables being the most produced crops, with 98% and 73%, respectively, and oilseeds being produced in smaller quantities. Out of the crops produced, families sold the most cereals and legumes (36.6%). The households reserved cereals and legumes for consumption and eventual sale, with corn, beans and cowpeas being the crops they reserved the most. The average annual duration of corn was 1.5±3.8 months and of beans 2.8±4.6 months. It was observed that 42% of the families had difficulties in growing vegetables, with insufficient seeds/seedlings being indicated as the main difficulty.

**Table 2-:** Nutritional and dietary status of children under 24 months (n = 284). Mouha, MN, Mozambique, 2023

**Classification of children’s nutritional status**										
Height/Age			Weight/Height				Weight/Age			
A*	L ^†^	M+G ^‡^	A	E ^§^	L ^†^	M+G ^‡^	A*	E ^§^	L ^†^	M+G ^‡^
156	75	53	176	76	23	9	180	42	53	9
55%	26%	19%	62%	27%	8%	3%	63%	15%	19%	3%
Adequacy of nutritional status in all indices										
0 to 5 months (n=80)			6 to 8 months (n=41)		9 to 11 months (n=38)		12 to 24 months (n=125)			
21 (26%)			17 (42%)		9 (24%)		26 (21%)			
**Dietary practices**										
Dietary adequacy in accordance with national recommendations										
0 to 5 months (n=80)			6 to 8 months (n=41)		9 to 11 months (n=38)		12 to 24 months (n=125)			
53 (67%)			2 (5%)		4 (11%)		7 (6%)			
Exclusive breastfeeding										
0 to 5 months (n=80)			6 to 8 months (n=41)		9 to 11 months (n=38)		12 to 24 months (n=125)			
53 (67%)			-		-		-			
Continued breastfeeding										
0 to 5 months (n=80)			6 to 8 months (n=41)		9 to 11 months (n=38)		12 to 24 months (n=125)			
-			36 (88%)		36 (95%)		110 (88%)			
Introduction of solid, semi-solid or soft foods										
0 to 5 months (n=80)			6 to 8 months (n=41)		9 to 11 months (n=38)		12 to 24 months (n=125)			
-			11 (27%)		-		-			
Minimum food diversity										
0 to 5 months (n=80)			6 to 8 months (n=41)		9 to 11 months (n=38)		12 to 24 months (n=125)			
-			6 (15%)		12 (30%)		30 (24%)			
Minimum meal frequency										
0 to 5 months (n=80)			6 to 8 months (n=41)		9 to 11 months (n=38)		12 to 24 months (n=125)			
-			23 (56%)		17 (45%)		74 (59%)			

*A = Adequate; ^†^L = Light; ^‡^M+G = Moderate + Severe; ^§^E = Excess

As for animal husbandry, families raised the most poultry, with emphasis on chickens (87%), cattle (44%) and, to a lesser extent, pigs. Of the species raised, cattle and goats were the most commercialized.

In Table 3, regarding access to food, production and sale were the main source of income for families. Even with the presence of production, 39.1% of families had difficulty in getting food during the year, lacking food, on average, for three months.

**Table 3-:** Food availability and access reported by household heads (n = 284). Mouha, MN, Mozambique, 2023

**Variables**	**Categories**	**Total**	**Percentage**
**Food availability indicators**			
Agricultural practice	Yes	281	99%
Food reserve	Yes	104	37%
Difficulty in cultivation	Yes	119	42%
Difficulty in feeding	Yes	111	39%
Crop variety	Leafy greens	206	72%
	Vegetables	90	32%
	Legumes	162	57%
	Cereals	279	98%
	Oilseeds	71	25%
Crops for sale	Legumes	122	43%
	Cereals	145	51%
Variety of animals	Cattle	125	44%
	Swine	5	2%
	Goat	109	38%
	Poultry	246	87%
Animals for sale	Cattle	125	44%
	Swine	5	2%
	Goat	109	38%
	Poultry	95	34%
Reservation duration	Corn*	1,5	3,8
	Beans*	2,8	4,6
**Food access indicators**			
Main source of income	Sale of production	193	68%
	Employment on a freelance basis	84	30%
	Food assistance/help/earnings/odd jobs	7	2%
Difficulties with eating during the year	≤3 months	91	32,0%
	≥4 months	22	7,7%
Food insecurity	Safety	99	35%
	Mild	89	31%
	Moderate	35	12%
	Severe	61	22%

*Mean and standard deviation

Of the total number of households, only 35% presented food security, 31% were mildly food security, 12% presented moderately food security, and 22% presented severely food insecurity, as shown in Table 3.

When all potential predictor variables were analyzed using nutritional status indicators as the outcome, the models that best fit were presented (weight/length or acute malnutrition and length/age or chronic malnutrition). It should be noted that the models obtained for the weight/age indicator were not prioritized because weight represented a generic measure of association between the height/age and weight/height indicators and were therefore considered less sensitive. Models were constructed for all children and for children aged 12 to 24 months who were most dependent on the family’s food reserves.

Having an older head of household appears as a protective factor against mild acute malnutrition, regardless of the child’s age. Among children aged 12 to 24 months, the head of household being male appears as a protective factor against mild acute malnutrition. The number of household members did not show an association with acute malnutrition but was negatively associated with mild and moderate chronic malnutrition, regardless of the child’s age. The total number of products for consumption was a protective factor against moderate and severe chronic malnutrition. In moderate and severe acute malnutrition among children aged 12 to 24 months, the variety of animals raised, starting with two animals, was a protective factor.

Having a greater quantity of products for sale was negatively associated with mild acute malnutrition among all children, and dietary adequacy appeared as a protective factor against moderate and severe acute malnutrition and moderate chronic malnutrition among all children, as shown in Table 4.

**Table 4-:** Multinomial log-linear models taking the weight/length index as the outcome variable, considering all children aged 12 to 24 months. Mouha, MN, Mozambique, 2023

Variables	Weight/length index			
	Light		Moderate+severe	
	Estimate	***p-value***	Estimate	***p-value***
Model 1 (all children, n=284)				
Age of the household head	0.41	0.022	0.23	0.396
Number of household members	-0.36	0.395	0.58	0.273
Total products for sale	-0.37	0.019	0.39	0.100
Children’s nutritional adequacy	0.71	0.159	1. 60	0.025
Model 2 (children from 12 to 24 months, n=125)				
Head of male household	16.00	<0.001	-2.00	0.172
Agricultural practice	17.00	<0.001	18.00	<0.001
Products for sale	-0.41	0.186	0.37	0.396
Animal breeding	0.58	0.664	4.00	<0.001
A variety of animals for consumption	-2.00	0.150	-8.00	<0.001
Two varieties of animals for consumption	-2.07	0.183	13.19	<0.001
Three varieties of animals for consumption	-0.89	0.572	14.00	<0.001
Difficulty feeding in household	-0.05	0.947	-68.00	<0,001

Being a male child represented a protective factor against moderate chronic malnutrition for all children. The age of the child from 6 to 11 months is associated with severe chronic malnutrition, and that of the child from 12 to 24 months old, with moderate chronic severe malnutrition, according to model 1. The presence of a man in the home (male head of the household) was a protective factor against severe chronic malnutrition for all children.

The practice of agriculture appears as a protective factor against acute malnutrition for children aged 12 to 24 months and as a protective factor against chronic malnutrition for all children in the moderate and severe classification and, in the model for children aged 12 to 24 months, in the mild, moderate and severe classifications of chronic malnutrition. Having products for sale showed no association with the outcome variables in any of the models. The number of cattle, in model 1, was negatively associated with mild chronic malnutrition, while in model 2, the number of pigs appeared as a protective factor in mild, moderate and severe malnutrition.

Feeding difficulties in the household were negatively associated with moderate and severe acute malnutrition and mild and moderate chronic malnutrition. In chronic malnutrition, in both models 1 and 2, the presence of food reserves was a protective factor in all classifications of chronic malnutrition (Table 5).

**Table 5-:** Multinomial log-linear models taking the length/age index as the outcome variable, considering all children aged 12 to 24 months old. Mouha, MN, Moçambique, 2023

Variables	Length/Age Index					
	Light		Moderate		Severe	
	Estim.*	***p- value***	Estim.*	***p- value***	Estim.*	***p-value***
Model 1 (all children, n=284)						
Male gender	0.587	0.058	11.00	0.016	0.58	0.285
6 to 8 months	-0.762	0.188	-0.75	0.541	2.00	<0.001
9 to 11 months	-0.006	0.991	0.44	0.657	18.00	<0.001
12 to 24 months	0.812	0.065	2.00	<0.001	19.00	<0.001
Male head of HH ^†‡^	0.542	0.692	24.00	0.074	3.00	0.041
Agricultural practice	-2.00	0.170	22.00	<0.001	11.00	<0.001
Food reserve	0.90	0.006	1.00	0.0150	1.00	0.022
Number of cattle	-0.07	0.047	-0.03	0.457	-0.02	0.702
Children’s nutritional adequacy	-0.17	0.703	-0.23	0.741	-19.00	<0.001
Feeding difficulties in HH ^‡^	0.49	0.113	0.94	0.032	0.16	0.779
Model 2 (children from 12 to 24 months, n=125)						
Number of members in the HH ^‡^	-0.92	0.033	-10.68	0.030	-0.43	0.397
Agricultural practice	28.32	<0.001	26.31	<0.001	10.00	<0.001
Total products for consumption	0.48	0.442	0.62	0.021	0.58	0.054
Food reserve	1.43	0.014	2.09	0.001	1.90	0.007
Number of pigs	29.00	<0.001	-9.08	<0.001	28.91	<0.001
Feeding difficulty in the HH ^‡^	1.44	0.010	2.20	<0.001	0.526	0.489

*Estim. = Estimate; ^†^Those who responded to the questionnaire intended for the head of the household; ^‡^HH = Household

In the adjustments for the weight-for-age indicator, the predictor variables were child age, gender of the household head and number of household members for all, and agricultural practice and dietary adequacy for children aged 12 to 24 months (data not shown).

## Discussion

In this study, it was observed that, in a region of Mozambique whose economy is based on the domestic food trade, where the persistence of child malnutrition had been drawing the attention of local authorities with a focus on food culture, malnutrition was associated not only with variables of feeding practices but also with local availability and access to food. Inadequate feeding practices for children were evidenced, however, the protective role of breastfeeding in the first year of life performed well. It was demonstrated that the existence of food reserves was more important than the existence of products for sale and it was also evidenced that, in households headed by men, children were less exposed to malnutrition. The most striking fact was that less than a third of the children evaluated were free from any type of nutritional problems assessed and that these problems were associated with all dimensions of FNS considered in the research, showing the complexity of the problem. Complementary feeding was analyzed in 80 countries^(23)^, based on national data sources, and it was observed that West and Central Africa presented the worst results regarding the diversity, frequency and adequacy of children’s food consumption and that only breastfeeding presented better rates in these locations.

Discussing inadequate feeding practices is a complicated task, starting by highlighting that these dietary traditions, passed down from mother to daughter, are still mechanisms of resilience in the face of an unfavourable food environment that has been perpetuated in the African continent^(1)^. In a previous study, in this same region of Mozambique, it was identified that the practice of breastfeeding was more frequently taught from mother to daughter than by health professionals^(24)^. In another study in the district of Sussundenga, it was observed how much patriarchy has been harmful to women, who lived overburdened with the tasks of caring for children and *muchambas* (small farms) and with little or almost no power to participate in local enterprises^(25)^. In the present study, 12% of households were headed by women, and the fact that the head of the household was a man and older than the others was a protective factor against malnutrition. This patriarchal characteristic has persisted in Africa since pre-colonial times^(26)^.

Exclusive breastfeeding in children under six months old was 67%, a significant value when considering that only 40% of children in the world are exclusively breastfed until six months of age^(27)^, although the ideal would be 80%. It was also noteworthy that almost all (97%) were on continued breastfeeding until one year. Studies indicate that the low coverage of exclusive breastfeeding is related to inadequate techniques and a lack of integrated communication and nutritional education activities^(28-29)^. In the present study, the appreciation of this traditional knowledge is very important, given the role of older and more experienced women in perpetuating this practice.

The child’s inadequate nutrition was clearly associated with severe and moderate acute malnutrition and severe chronic malnutrition (Tables 4 and 5). When we looked for an association with age and chronic malnutrition, we found a negative association within the nutritional transition age groups (6 to 8 months old and 9 to 11 months old) and a positive association among children older than one year (Table 5), reinforcing the importance of the nutritional transition period that occurs between six and 11 months, when children gradually become less dependent on breast milk. In addition, it is worth noting the behavior of the data on nutritional status adequacy for all indicators, which were low up to five months of age, higher at six and eight months, and returning to initial levels between the ages of nine and 24 months. We do not have information on the birth weight of these children, which may have influenced the inadequate nutritional status in the first months of life. It is likely that greater adequacy from six to eight months was benefited by exclusive breastfeeding at the beginning of life and that, little by little, this gain was compromised by the inadequacy of the dietary transition. These findings corroborate the evidence that complementary feeding should begin at six months, with a variety of foods and adequate frequency, ensuring breastfeeding until 24 months^(30-32)^.

Traditionally, the basis of the meals of less privileged children has been composed of cereals, because this is what the environment has produced and accessed^(3,33-34)^. Fortified baby food has been recommended as an alternative^(25)^, so the question arises: why do mothers insist on not adhering to this recommendation of enriching baby food? This situation deserves reflection in view of the fact that the practice of agriculture is a protective factor against malnutrition. Farmers who managed to obtain food reserves had their children protected from malnutrition. Although these reserves were mainly based on corn and beans, the protected children also had access to a variety of animals and vegetables for consumption.

There is already sufficient evidence on the importance of dietary diversity for maintaining good nutritional status, especially from the moment when the dietary transition is carried out, which should include the consumption of fruits and vegetables^(35-36)^. It seems that the best path forward is to invest in strengthening farmers and agriculture, deepening knowledge of local practices and the real needs of those who do not produce enough (seeds, seedlings, baby animals, other inputs and social technologies).

It was observed that not all households sold what they produced and, more than selling, producing enough to have food reserves was what allowed them to avoid difficulties with feeding and, consequently, to have well-nourished children. Having a variety of animals for consumption was what protected against chronic malnutrition. In acute malnutrition, cattle raising appeared negatively associated with protection from acute malnutrition for all children and pig raising protected children over one year old. In addition, more than half of the animal reared in households was composed of poultry, also used for consumption. This reinforces the importance of having food for consumption, since selling it (in the case of cattle) did not help protect against malnutrition. The difficulty with feeding the family mainly affected children over one year old, whether in the acute or chronic malnutrition indicator. These older children were less protected by breastfeeding.

According to the Mozambique livestock statistics bulletin, there was an increase in cattle production in 2022 of approximately 5% compared to 2021, including family production^(37)^. As for cattle, since it is chosen for commercialization, it is assumed that families have the possibility of earning money for self-sustainability by selling it. It seems, however, that, given the fact that the production of small animals is the one that presents itself as a protective factor against malnutrition, the incentive for livestock farming should come in the sense of supporting the production of small animals as a source of protein in the diet of households. Nevertheless, the issue is complex, as these families have needs that need to be met, in addition to food, including foodstuffs that cannot be produced locally. The sale of food and livestock (68%) was identified as the main source of income for these families, which tend to be large and the larger the family, the greater the presence of mild and moderate chronic malnutrition among children over one year old, who are more dependent on the family’s food reserves. It was observed that 65% of the households presented some degree of food insecurity.

With regard to public policies, the Mozambican government rightly prioritized exclusive breastfeeding in the National Infant Feeding Strategy in 2019^(38-39)^. Furthermore, with a view to responding to Sustainable Development Goals 1 and 2, inspired by the Food and Nutrition Security Strategy of the Community of Portuguese-Speaking Countries (ESAN-CPLP)^(40)^, it has been following the guidelines of the Food and Nutrition Security Council of the Community of Portuguese-Speaking Countries (CONSAN-CPLP)^(41)^ and the voluntary guidelines of the FAO^(42)^, seeking to eradicate hunger and promote sustainable agriculture, reinforcing existing public policies from the perspective of the human right to adequate food and sustainability. To this end, it has formulated and implemented several action plans, with particular emphasis on the Strategic Plan for the Development of the Agricultural Sector^(43)^. Through the pillar of production, productivity and agrarian competitiveness, actions were established to ensure FNS supported by the promotion of food consistency and accessibility; investments in the expansion of physical food reserves; establishment and/or strengthening and operationalization of social protection programs that ensure access to food banks for vulnerable communities; strengthening the implementation and/or establishment of strategic food fortification programs, including biofortification; promotion of social and behavioral change with a view to diversifying and balancing the diet; and strengthening the monitoring and regular evaluation of FNS. This shows the concern of the Mozambican government with FNS; however, the implementation of these policies has not yet been reflected in the households of Mouha and their children. Here, a discussion of the structural and global causes that affect the African continent, diplomatic cooperation relations, among other historically perpetuated inequities would be appropriate^(44)^.

Considering the complexity of determining child malnutrition, we can point out, as a limitation of this study, that it did not consider other health determinants, such as housing conditions, sanitation, access to services and protective care. Care was taken to select children from health units without any diagnosis of disease associated with malnutrition, and it was considered that the opportunities for access to services were the same for the entire population, which gives validity to the results presented. This study points to a path to be explored for understanding local dynamics and strengthening these farmers, and especially women farmers, so that they can obtain sustenance for their households and be able to adequately care for their children, and also the need for advancing knowledge that will contribute to more effective public policies.

## Conclusion

Malnutrition in children under 24 months old, whether acute or chronic, is also associated with adequate feeding practices, availability of food in the local environment and access to it for consumption.

## Data Availability

All data generated or analysed during this study are included in this published article.
