# Supplementary material for: Availability, access and feeding practices associated with malnutrition in children under 24 months in Mozambique
Source: Rev Lat Am Enfermagem. 2025 Nov 3;33:e4602. doi: 10.1590/1518-8345.7366.4602 (PMC12587755; doi:10.1590/1518-8345.7366.4602)
Supplement: Translation

# Translation: Disponibilidade, acesso e práticas alimentares associados à desnutrição em crianças com menos de 24 meses em Moçambique*

**DOI:** 10.1590/1518-8345.7366.4603

**Authors:** Florência Custódio Mondlane, Lara Morena Cardeal, Rogério António de Oliveira, Maria Rita Marques de Oliveira

**Affiliations:** 1 Universidade Estadual Paulista, Faculdade de Medicina, Botucatu, SP, Brasil.; 2 Bolsista da Coordenação de Aperfeiçoamento de Pessoal de Nível Superior (CAPES), Brasil.; 3 Universidade Estadual Paulista, Instituto de Biociências, Botucatu, SP, Brasil.; 4 Bolsista do Conselho Nacional de Desenvolvimento Científico e Tecnológico (CNPq), Brasil.

**Keywords:** Nutrição da Criança, Desnutrição, Aleitamento Materno, Segurança Alimentar, Produção de Alimentos, Práticas Alimentares

## Abstract

analisar a associação da desnutrição com a disponibilidade, o acesso e as práticas alimentares de crianças com menos de 24 meses em Mouha, Moçambique.

estudo observacional do tipo transversal analítico, com amostra de 284 crianças com menos de 24 meses, cujos chefes de família e mães ou responsáveis pela alimentação da criança responderam, no domicílio, a formulários estruturados. Utilizaram-se modelos *log*-lineares multinominais para verificar a associação das variáveis preditoras com indicadores antropométricos de desnutrição.

foi observado que menos de um terço das crianças estava livre da desnutrição. Apenas 23% das crianças apresentaram adequação alimentar que atendia a todos os critérios para sua faixa etária, a qual foi negativamente associada a desnutrição aguda e crônica grave; 67% das crianças recebiam aleitamento materno exclusivo, e a prevalência de aleitamento continuado ocorria entre 88% e 95%. A existência de reserva alimentar foi fator protetor à desnutrição crônica em todos os graus, confirmada pela prática de agricultura e pela diversidade de animais criados. O problema alimentar esteve associado a desnutrição aguda e crônica nas crianças maiores de um ano.

a desnutrição de crianças com menos de 24 meses associa-se à adequação alimentar, à disponibilidade dos alimentos para o consumo e ao acesso a eles.

## Destaques

**(1)** Os agregados familiares são compostos predominantemente por agricultores. **(2)** A desnutrição é o resultado de práticas alimentares inadequadas. **(3)** Crianças com mais de um ano estavam menos protegidas da desnutrição. **(4)** Menos de um terço das crianças estava livre da desnutrição.

## Introdução

O direito humano à alimentação adequada consta nas principais pautas internacionais e representa um dos mais desafiadores objetivos do desenvolvimento sustentável, guardando interdependência com todos os outros. A fome, a desnutrição e outras formas de má nutrição têm atingido mais que a metade da população mundial. Em 2022, a fome atingia entre 691 e 783 milhões de pessoas no mundo, segundo estimativas das Nações Unidas. Embora tenham sido feitos alguns progressos em outras regiões afetadas, a fome continuou a aumentar em todas as regiões da África, atingindo principalmente os habitantes das áreas rurais e domicílios sob a responsabilidade de mulheres^(1)^.

No mundo todo, mais de 22,3% das crianças apresentavam déficit de crescimento em 2022, sendo que 6,8% apresentavam magreza e 5,6% estavam acima do peso esperado. Em Moçambique, eram 2 milhões de crianças com déficit de crescimento, o que representava aproximadamente 5,3% da sua população total^(1)^. Estimou-se que a prevalência da desnutrição crônica em idade pré-escolar no país era de 37%^(2)^. Esse quadro vem sendo agravado pelo acesso inadequado à água e ao saneamento, principalmente na região norte do país^(2)^. A boa nutrição nos primeiros anos de vida é primordial para o desenvolvimento e crescimento pleno das crianças com vistas a garantir uma vida plena e saudável até a fase adulta^(3)^.

As medidas antropométricas e seus índices derivados são usados como indicadores do estado nutricional, sendo de fácil obtenção e sensíveis às alterações da composição corporal, amplamente utilizados para avaliar e monitorar a saúde nutricional da população^(4-5)^. A insuficiência proteico-calórica recente associa-se à magreza e ao baixo peso, e a emaciação pode significar desnutrição aguda; por outro lado, a carência constante desses nutrientes, por longos períodos, resulta em comprometimento do desenvolvimento infantil, denunciado pela baixa estatura ou desnutrição crônica^(6-7)^.

A desnutrição materno-infantil apresenta consequências em curto e longo prazo. A morbimortalidade e o comprometimento do desenvolvimento físico e psicossocial apresentam-se como consequências em curto prazo, resultando no aumento da mortalidade infantil e da suscetibilidade às doenças. Em longo prazo, a desnutrição pode resultar em dificuldades cognitivas, piora na performance reprodutiva, incidência de excesso de peso e doenças metabólicas na vida adulta como consequência da fome enquanto criança^(8)^.

A desnutrição tem sido avaliada a partir de causas imediatas, subjacentes e básicas: a ingestão inadequada de alimentos e as doenças estão entre as causas imediatas da desnutrição, enquanto a insegurança alimentar e a inadequação do cuidado e da atenção à saúde estão entre as causas subjacentes. Já como causas básicas, constam a falta de acesso às políticas públicas (de saúde, educação, trabalho, fundiária) e às tecnologias, condicionadas ao insuficiente capital financeiro, humano e às condições físicas e sociais do contexto em que se vive^(9)^.

A Segurança Alimentar e Nutricional (SAN) é um fator preponderante na garantia do bom estado nutricional. A SAN existe quando todas as pessoas, em todos os momentos, têm acesso físico, social e econômico a alimentos seguros, nutritivos e suficientes que atendam às suas necessidades dietéticas e preferências alimentares para uma vida ativa e saudável, embasada nas práticas alimentares promotoras de saúde, respeitando a diversidade cultural, ou seja, ambiental, cultural, econômica e socialmente sustentáveis^(10)^. Essa concepção envolve a dimensão da disponibilidade dos alimentos no contexto ambiental; o acesso físico e financeiro no contexto familiar; o uso no contexto biológico, afetivo e cultural; a estabilidade e sustentabilidade dos três primeiros e o poder de ação de direito do sujeito^(10)^.

Os estudos têm revelado que a agropecuária familiar para o autoconsumo contribui positivamente com a saúde e o sustento das famílias e suas crianças e com a redução da insegurança alimentar^(11)^. No entanto, diante da complexidade do processo de determinação social da desnutrição, surge a necessidade de aprofundar o seu entendimento, levando em conta questões culturais, sociais e ambientais, seja dos meios de subsistência, seja das práticas alimentares^(12)^.

No caso das crianças, o papel da mãe ou cuidador é muito relevante. Foi observado, por exemplo, que o aleitamento materno exclusivo entre crianças de 0 a 5 meses e a adequada transição nutricional eram mais prejudicados em crianças cujas mães trabalhavam fora de casa^(13)^; além disso, destaca-se também que em Moçambique as dietas são, na sua maioria, à base de cereais, o que tem limitado a diversidade alimentar de crianças de 6 a 24 meses^(14)^.

Mouha é uma localidade do distrito de Sussundenga, na província de Manica em Moçambique, uma região que foi muito afetada por eventos climáticos, mas onde mais se produz alimentos no país; no entanto, é também uma das regiões mais afetadas pela desnutrição infantil^(15)^. Foi postulado que a desnutrição infantil na região pode ser decorrente de práticas alimentares inadequadas exercidas pelas mães ou responsáveis pela alimentação da criança. Na literatura referente aos países africanos de língua portuguesa, as informações existentes sobre a desnutrição correspondem a dados de estudos transversais que pouco se aprofundam nas causas da desnutrição^(16)^; não se sabe ainda se as crianças afetadas pela desnutrição estão submetidas a baixa disponibilidade de alimento e pouco acesso a ele ou se as práticas alimentares são inadequadas. Diante da complexidade do processo de determinação da desnutrição e da falta de dados da região, nesta pesquisa partiu-se da hipótese de que variáveis relacionadas à determinação do estado nutricional relativas à disponibilidade, ao acesso e consumo poderiam estar produzindo a desnutrição infantil em Mouha, como provavelmente em outras localidades com características semelhantes. Assim, o objetivo desta pesquisa foi analisar a associação da desnutrição com a disponibilidade, o acesso e as práticas alimentares de crianças com menos de 24 meses em Mouha, Moçambique.

## Método

### Tipo de estudo

Trata-se de um estudo observacional do tipo transversal analítico que envolveu a coleta de informações por meio de inquérito domiciliar, sendo que, para redação deste documento, foi seguido o *checklist* STROBE (*Strengthening the Reporting of Observational Studies in Epidemiology*).

### População e local de estudo

A população foi constituída por crianças com menos de 24 meses, o chefe da família e a mãe ou o responsável pela alimentação da criança, residentes na localidade de Mouha, distrito de Sussundenga, província de Manica em Moçambique, fossem eles praticantes ou não da agricultura e pecuária. A economia do distrito é baseada na agricultura e a localidade é composta por 14 bairros, uma unidade sanitária do tipo II (unidade sanitária pequena que dispensa cuidados de saúde primários em meio rural) e quatro escolas primárias.

### Critério de seleção

Foram convidados os chefes de família, as mães ou responsáveis pela alimentação de criança com menos de 24 meses cadastrada na unidade sanitária (US), independentemente do tempo de residência na localidade, que não apresentasse problemas de saúde que afetassem diretamente o estado nutricional (HIV/SIDA, tuberculose, atraso no desenvolvimento psicomotor), conforme indicado no cartão de saúde da criança ou ficha pré-natal. Excluíram-se do estudo todos os Agregados Familiares (AF) (essa é uma designinação específica, com legislação própria, para um agrupamento familiar em Moçambique, não usual no Brasil) que não completaram os dados da pesquisa e/ou crianças diagnosticadas *aposteriori* com problemas que afetassem diretamente o estado nutricional.

### Amostra e amostragem

Empregou-se um plano de amostragem aleatória simples por conglomerados, com o poder de 80% e o nível de significância de 5%, considerando-se 22 variáveis independentes a serem analisadas por regressão linear múltipla^(17)^, o que resultou em 234 crianças em seus respectivos AFs, sendo apenas uma criança de cada AF. Foi considerado 20% de perda (n=340), sendo que, no final, foram incluídos na pesquisa 284 AFs provenientes de 10 dos 14 bairros existentes. Primeiramente, foram contactados os líderes e/ou secretários para, na sequência e com a devida autorização, convidar as famílias com crianças com menos de 24 meses a participarem da pesquisa.

### Instrumentos utilizados e variáveis do estudo

Foram usados dois formulários estruturados para coleta de dados, um aplicado ao chefe do AF ou seu representante e o outro à mãe ou responsável pela alimentação da criança. O formulário dirigido ao chefe do AF era composto pelas seções: A - com questões relativas a dados demográficos do chefe do AF; seção B - com questões relativas à produção de cultura e pecuária; seção C - com perguntas sobre as formas de vida; seção D - com questões de consumo alimentar, incluindo o recordatório de 24 h e o recordatório de uma semana; seção E - com questões sobre avaliação da insegurança alimentar em que foi aplicada a FIES - *Food Insecurity Experience Scale*, desenvolvida pela *Food and Agriculture Organization* (FAO) como feramenta global, incluindo a validação para Moçambique^(18)^.

O segundo formulário foi dirigido à mãe ou responsável pela alimentação da criança, composto por seções A e B; na seção A, as questões eram sobre dados demográficos e antropométricos das crianças, e na seção B, questões sobre aleitamento materno exclusivo, aleitamento continuado e alimentação complementar em formato de recordatório de 24 h e de uma semana dos grupos de alimentos consumidos.

Ambos os formulários foram obtidos a partir do instrumento de coleta de dados do Secretariado Técnico de Segurança Alimentar e Nutricional (versão 21/02/2018)^(19)^, compostos de respostas predefinidas, tais como: sim, não, não sabe, respostas curtas e respostas de escolha múltipla correspondentes à realidade do participante.

Os alimentos foram agrupados de acordo com a classificação de alimentos da FAO, conforme segue: cereais; vegetais e tubérculos ricos em vitamina A; vegetais e folhas verde-escuras; outros vegetais; leguminosas; frutas de cor alaranjada ou amareladas; outras frutas; carnes (brancas e vermelhas); ovos; leite e produtos lácteos; peixes e frutos do mar; vísceras; sementes; óleos e gorduras; doces e açúcar; temperos e condimentos^(20)^.

### Variáveis do estudo

As variáveis do estudo foram organizadas em quatro dimensões:

Variáveis demográficas: 1) Idade do chefe do AF; 2) Sexo do chefe do AF; 3) Nível de escolaridade do chefe do AF; 4) Número de membros no AF; 5) Sexo da criança; 6) Número de crianças com menos de 24 meses em cada A; 7) Idade da criança.

Estado nutricional: 8) Peso/Idade; 9) Peso/Estatura; 10) Estatura/Idade; 11) Presença de edema bilateral (nenhum caso constatado).

Disponibilidade alimentar: 12) Prática ou não da agricultura; 13) Variedade de cultivo, considerando verduras (couve, alface, amaranto, folha de abóbora, outro); legumes (cenoura, tomate, pimenta, quiabo, repolho, outro); leguminosas (feijões, amendoim, outro); cereais (milho, sorgo, trigo, arroz, outro); oleaginosas (girassol, gergelim, coco, outro); 14) Culturas para venda: verduras (couve, alface, amaranto, folha de abóbora, outro); legumes (cenoura, tomate, pimenta, quiabo, repolho, outro); leguminosas (feijões, amendoim, outro); cereais (milho, sorgo, trigo, arroz, outro); oleaginosas (girassol, gergelim, coco, outro); 15) Duração da reserva; 16) Dificuldades de cultivo; 17) Variedade de animais; 18) Animais para venda; 19) Adequação alimentar.

Acesso alimentar: 20) Principal fonte de renda: produção, venda de culturas e animais; trabalho por conta própria (processamento, salário, pensões, remessas, comércio, serviços) e assistência alimentar/ajuda/ganho/trabalho eventual; 21) Dificuldades de alimentação, número de meses com dificuldades de alimentação; 22) Insegurança alimentar, práticas alimentares; 23) Adequação às recomendações nacionais; 24) Aleitamento materno exclusivo; 25) Aleitamento materno continuado; 26) Introdução de alimentos sólidos, semissólidos ou moles; 27) Diversidade alimentar mínima; 28) Frequência mínima de refeições.

Importa esclarecer que foi considerada como “sim” para adequação alimentar aquela criança que, consoante a idade, recebe alimentação adequada de acordo com as recomendações da Organização Mundial da Saúde (OMS), do Fundo das Nações Unidas para a Infância (UNICEF) e da FAO (adotadas pelo Ministério de Saúde de Moçambique), sendo que de 0 a 5 meses deve receber o aleitamento materno exclusivo, de 6 a 24 meses deve ter aleitamento continuado, ter diversidade alimentar mínima e um número mínimo de refeições, conforme a idade.

Para análise da Insegurança Alimentar, foram utilizados o questionário e *template* para análise em *Excel* ^®^ fornecidos pela FAO^(21)^, que contemplam instrumentos validados para o português de Moçambique e em dois dialetos. As variáveis demográficas, do estado nutricional, da disponibilidade alimentar e do acesso alimentar foram consideradas como variáveis independentes, e as de desfecho foram os índices nutricionais. Para efeito de análise, a idade do chefe do AF; o tempo de escolaridade do chefe do AF; o número de membros no AF; a idade da criança; o número de crianças com menos de 24 meses no AF, e o total de meses com dificuldades de alimentação foram estratificados por categorias.

### Coleta de dados e período

A coleta foi antecedida por um estudo-piloto para avaliar a percepção das questões e interpretação dos dados e por uma breve apresentação da pesquisadora à chefe da localidade e aos chefes dos bairros. A coleta teve início em 16 de janeiro de 2023, no sentido do bairro mais próximo da sede da localidade ao mais distante e do domicílio mais distante ao mais próximo, com exceção de um dos bairros mais próximos da sede, que foi um dos últimos a ser visitado por falta de meios de comunicação com a liderança local. Primeiramente o líder do bairro informava as famílias sobre a pesquisa que seria desenvolvida e as datas previstas para coleta de dados em cada bairro e, na sequência, a pesquisadora dirigia-se ao domicílio de cada AF, acompanhada do líder do bairro ou não. A média de duração da entrevista com cada chefe ou representante dos agregados familiares foi de 20 minutos e de 40 minutos com as mães ou responsáveis pela alimentação das crianças, incluindo a avaliação antropométrica.

### Questões éticas

Iniciou-se a pesquisa após a aprovação do Comitê Nacional de Bioética para Saúde do Ministério da Saúde de Moçambique, na Ref. 882/CNBS/22 e a obtenção do Termo de Consentimento Livre e Esclarecido (TCLE) para participantes maiores e de assentimento para mães ou responsáveis pela alimentação das crianças que fossem menores de 18 anos. Toda informação foi destruída após ser transcrita, analisada e interpretada, salientando-se que os dados foram anonimizados para a análise.

### Análise dos dados

Os dados coletados foram organizados em uma planilha eletrônica. Posteriormente, com o intuito de identificar padrões e compreender a natureza dos dados, foram realizadas análises descritivas utilizando a linguagem de programação estatística R, pacote *dplyr* ^(22)^. As proporções obtidas como resultados para as variáveis qualitativas e quantitativas foram organizadas em tabelas, sendo que as variáveis demográficas, de disponibilidade alimentar (com exceção da variável duração da reserva que foi expressa em média) e acesso alimentar foram categorizadas e expressas em percentual.

Para investigar a associação entre as variáveis desfecho e preditoras, os dados foram divididos para a realização das análises. A primeira análise foi realizada ajustando modelos *log*-lineares multinominais, considerando os dados de todas as crianças em meses. De forma similar, o segundo ajuste de modelo *log*-linear multinominal foi realizado, considerando-se os dados que possuem categoria de idade da criança com o filtro de 12 a 24 meses. Nesse contexto, consideramos cada índice nutricional como variável desfecho, com o objetivo de determinar quais variáveis preditoras tiveram maior impacto nas variáveis desfecho, considerando-se 5% de significância.

## Resultados

Das 284 famílias estudadas, a maior parte dos chefes dos AFs tinha idade ≥ 41 anos (34%). Do total, 88% eram do sexo masculino, 51% tinham sete anos de estudos e 4% não haviam frequentado a escola; mais de 50% dos AFs eram compostos por seis ou mais membros e, na maioria (96%), existia apenas uma criança com menos de 24 meses. Do total de crianças, 51% eram do sexo masculino e a maior parte (44%) apresentava idade de 6 a 24 meses (Tabela 1).

**Tabela 1-:** Dados demográficos das famílias (n = 284). Mouha, MN, Moçambique, 2023

**Variáveis**	**Categorias**	**Total**	**Percentual**
Idade do chefe do AF* (em anos)	≤ 20	3	1%
	21-25	55	19%
	26-30	55	19%
	31-35	41	14%
	36-40	34	12%
	≥ 41	96	35%
Sexo do chefe do AF*	Masculino	250	88%
	Feminino	34	12%
Nível de escolaridade do chefe do AF* (em anos)	Nenhum estudo	12	4%
	Até 7 anos de estudo	127	45%
	Mais de 7 anos de estudo	145	51%
Número de membros no AF*	≤ 5	132	47%
	6 a 10	130	46%
	≥ 11	22	7%
Sexo da criança	Masculino	144	51%
	Feminino	140	49%
Crianças com menos de 24 meses em cada AF*	1	273	96%
	≥ 2	11	4%
Idade da criança (em meses)	0 a 5	80	29%
	6 a 8	41	14%
	9 a 11	38	13%
	12 a 24	125	44%

*AF = Agregado familiar

Das 284 crianças avaliadas, 51% classificaram-se com comprimento adequado para a idade, 60% com o peso adequado para o comprimento e 63% com o peso adequado para a idade. No entanto, quando se considerou o conjunto de indicadores com as crianças distribuídas por faixa etária, a proporção de crianças com todos os índices adequados foi inferior, variando de 42%, entre as de 6 a 8 meses, a 21%, entre as de 12 a 24 meses, conforme a Tabela 2.

**Tabela 2-:** Estado nutricional e alimentar das crianças com menos de 24 meses (n = 284). Mouha, MN, Moçambique, 2023

**Classificação do estado nutricional das crianças**										
Comprimento/Idade			Peso/Comprimento				Peso/Idade			
A*	L ^†^	M+G ^‡^	A	E ^§^	L ^†^	M+G ^‡^	A*	E ^§^	L ^†^	M+G ^‡^
156	75	53	176	76	23	9	180	42	53	9
55%	26%	19%	62%	27%	8%	3%	63%	15%	19%	3%
Adequação do estado nutricional em todos os índices										
0 a 5 meses (n=80)			9 a 11 meses (n=38)				12 a 24 meses (n=125)			
21 (26%)			9 (24%)				26 (21%)			
**Práticas alimentares**										
Adequação alimentar de acordo com as recomendações nacionais										
0 a 5 meses (n=80)			9 a 11 meses (n=38)				12 a 24 meses (n=125)			
53 (67%)			4 (11%)				7 (6%)			
Aleitamento materno exclusivo										
0 a 5 meses (n=80)			9 a 11 meses (n=38)				12 a 24 meses (n=125)			
53 (67%)			-				-			
Aleitamento materno continuado										
0 a 5 meses (n=80)			9 a 11 meses (n=38)				12 a 24 meses (n=125)			
-			36 (95%)				110 (88%)			
Introdução de alimentos sólidos, semissólidos ou moles										
0 a 5 meses (n=80)			9 a 11 meses (n=38)				12 a 24 meses (n=125)			
-			-				-			
Diversidade mínima alimentar										
0 a 5 meses (n=80)			9 a 11 meses (n=38)				12 a 24 meses (n=125)			
-			12 (30%)				30 (24%)			
Frequência mínima de refeições										
0 a 5 meses (n=80)			9 a 11 meses (n=38)				12 a 24 meses (n=125)			
-			17 (45%)				74 (59%)			

*A = Adequado; ^†^L = Leve; ^‡^M+G = Moderado + Grave; ^§^E = Excesso

Quanto à prática alimentar, ao ser analisada a adequação conforme o que preconizam as diretrizes adotadas no país para cada faixa etária, apenas 66 (23%) das crianças atendiam a todos os critérios. Entre as crianças de 0 a 5 meses, 67% recebiam aleitamento materno exclusivo; entre as de 6 a 8 meses, 88% estavam em aleitamento continuado; 56% tinham frequência mínima de refeições; e 15% apresentavam diversidade alimentar mínima. Na faixa etária de 9 a 11 meses, 95% estavam em aleitamento continuado; 30% e 45% tinham diversidade alimentar mínima e frequência mínima de refeições, respectivamente. Das 125 crianças de 12 a 24 meses, 88% estavam em aleitamento continuado; 24% tinham diversidade alimentar mínima e 59% apresentavam frequência mínima de refeições (Tabela 2).

O levantamento da disponibilidade alimentar nos AFs mostrou que a maioria das famílias praticava a agricultura, sendo que os cereais e as verduras eram as culturas mais produzidas, com 98% e 73%, respectivamente, e as oleaginosas eram produzidas em menor quantidade. Das culturas produzidas, as famílias comercializavam mais os cereais e leguminosas (36,6%). Os AFs reservavam cereais e leguminosas para consumo e eventual venda, sendo o milho, o feijão catarina e o feijão *nhemba* as culturas que mais reservavam. A duração anual média do milho era de 1,53,8 meses e dos feijões de 2,84,6 meses. Foi observado que 42% das famílias apresentavam dificuldades para cultivo de vegetais, sendo que a insuficiência de sementes/mudas foi apontada como principal dificuldade.

Quanto à criação de animais, as famílias criavam mais aves, com destaque para as galinhas (87%), gado bovino (44%) e, em menor quantidade, os suínos. Das espécies criadas, o gado bovino e caprino eram os mais comercializados.

Na Tabela 3, sobre o acesso ao alimento, a produção e venda eram a principal fonte de renda das famílias. Mesmo com a presença de produção, 39,1% das famílias apresentavam dificuldade de alimentação durante o ano, faltando comida, em média, por mais de três meses.

**Tabela 3-:** Disponibilidade de alimento e acesso a ele referidos pelos chefes do AF (n = 284). Mouha, MN, Moçambique, 2023

**Variáveis**	**Categorias**	**Total**	**Percentual**
**Indicadores de disponibilidade alimentar**			
Prática da agricultura	Sim	281	99%
Reserva alimentar	Sim	104	37%
Dificuldade de cultivo	Sim	119	42%
Dificuldade alimentação	Sim	111	39%
Variedade de cultivo	Verduras	206	72%
	Legumes	90	32%
	Leguminosas	162	57%
	Cereais	279	98%
	Oleaginosas	71	25%
Culturas para venda	Leguminosas	122	43%
	Cereais	145	51%
Variedade de animais	Bovino	125	44%
	Suíno	5	2%
	Caprino	109	38%
	Aves	246	87%
Animais para venda	Bovino	125	44%
	Suíno	5	2%
	Caprino	109	38%
	Aves	95	34%
Duração da reserva	Milho*	1,5	3,8
	Feijões*	2,8	4,6
**Indicadores de acesso ao alimento**			
Principal fonte de renda	Venda da produção	193	68%
	Trabalho por conta	84	30%
	Assistência alimentar/ajuda/ganho/trabalho eventual	7	2%
Dificuldades com a alimentação no ano	≤ 3 meses	91	32,0%
	≥ 4 meses	22	7,7%
Insegurança alimentar	Segurança	99	35%
	Leve	89	31%
	Moderada	35	12%
	Grave	61	22%

*Média e desvio-padrão

Do total de AFs, apenas 35% apresentavam segurança alimentar, 31% insegurança alimentar leve, 12% insegurança alimentar moderada e 22% insegurança alimentar grave, conforme a Tabela 3.

Quando todas as variáveis potenciais preditoras foram analisadas tomando os indicadores do estado nutricional como desfecho, os modelos que melhor se ajustaram foram apresentados (peso/comprimento ou desnutrição aguda e comprimento/idade ou desnutrição crônica), destacando-se que os modelos obtidos para o indicador peso/idade não foram priorizados porque o peso representava uma medida genérica de associação dos indicadores estatura/idade e peso/estatura, tendo sido considerados, portanto, menos sensíveis. Foram construídos modelos para todas as crianças e para crianças de 12 a 24 meses mais dependentes das reservas alimentares da família.

Ter um chefe do AF mais velho apareceu como fator protetor na desnutrição aguda leve, independentemente da idade da criança. Entre as crianças de 12 a 24 meses, o chefe do AF ser do sexo masculino apareceu como fator protetor na desnutrição aguda leve. O número de membros do AF não mostrou associação com a desnutrição aguda, mas associou-se negativamente com a desnutrição crônica leve e moderada independentemente da idade da criança. Já o total de produtos para o consumo foi fator protetor na desnutrição crônica moderada e grave. Na desnutrição aguda moderada e grave das crianças de 12 a 24 meses, a variedade de animais criados, a partir de dois animais, foi fator protetor.

Apresentar uma maior quantidade de produtos para venda associou-se negativamente à desnutrição aguda leve entre todas as crianças, e a adequação alimentar apareceu como fator protetor da desnutrição aguda moderada e grave e da desnutrição crônica moderada entre todas as crianças, conforme mostra a Tabela 4.

**Tabela 4-:** Modelos *log-*lineares multinominais tomando o índice peso/comprimento como variável de desfecho, considerando todas as crianças e aquelas de 12 a 24 meses. Mouha, MN, Moçambique, 2023

Variáveis	Índice peso/comprimento			
	Leve		Moderado+grave	
	Estimativa	***p-valor***	Estimativa	***p-valor***
Modelo 1 (todas as crianças, n=284)				
Idade do chefe do AF*	0,41	0,022	0,23	0,396
Número de membros no AF*	-0,36	0,395	0,58	0,273
Total de produtos para venda	-0,37	0,019	0,39	0,100
Adequação alimentar da criança	0,71	0,159	1, 60	0,025
Modelo 2 (crianças de 12 a 24 meses, n=125)				
Chefe do AF* do sexo masculino	16,00	<0,001	-2,00	0,172
Prática da agricultura	17,00	<0,001	18,00	<0,001
Produtos para venda	-0,41	0,186	0,37	0,396
Criação de animais	0,58	0,664	4,00	<0,001
Uma variedade de animais para consumo	-2,00	0,150	-8,00	<0,001
Duas variedades de animais para consumo	-2,07	0,183	13,19	<0,001
Três variedades de animais para consumo	-0,89	0,572	14,00	<0,001
Dificuldade de alimentação no AF*	-0,05	0,947	-68,00	<0,001

*AF = Agregado familiar

A criança ser do sexo masculino representou fator protetor na desnutrição crônica moderada para todas as crianças. A idade da criança de 6 a 11 meses está associada à desnutrição crônica grave, e a de 12 a 24 meses, à moderada crônica grave, conforme modelo 1. A presença do homem em casa (chefe do AF masculino) foi fator protetor na desnutrição crônica grave de todas as crianças.

A prática da agricultura aparece como fator protetor na desnutrição aguda das crianças de 12 a 24 meses e como fator protetor da desnutrição crônica de todas as crianças na classificação moderada e grave e, no modelo para as crianças de 12 a 24 meses, nas classificações leve, moderada e grave da desnutrição crônica. Ter produtos para a venda não mostrou associação com as variáveis de desfecho em nenhum dos modelos.

A quantidade de gado bovino, no modelo 1, associou-se negativamente com a desnutrição crônica leve, já no modelo 2 a quantidade de suínos apareceu como fator protetor na desnutrição leve, moderada e grave.

A dificuldade de alimentação no AF associou-se negativamente à desnutrição aguda moderada e grave e à desnutrição crônica leve e moderada. Na desnutrição crônica, tanto no modelo 1 quanto no modelo 2, a presença de reserva alimentar foi fator de proteção em todas as classificações da desnutrição crônica (Tabela 5).

Nos ajustes para o indicador peso para idade, as variáveis preditoras foram idade da criança, sexo do chefe do AF e número de membros no AF para todos, e prática de agricultura e adequação da alimentação para crianças de 12 a 24 meses (dados não mostrados).

**Tabela 5-:** Modelos *log*-lineares multinominais tomando o índice comprimento/idade como variável de desfecho, considerando todas as crianças e aquelas de 12 a 24 meses. Mouha, MN, Moçambique, 2023

Variáveis	Índice comprimento/idade					
	Leve		Moderado		Grave	
	Estim.*	***p- valor***	Estim.*	***p- valor***	Estim.*	***p-valor***
Modelo 1 (todas as crianças, n=284)						
Sexo masculino	0,587	0,058	11,00	0,016	0,58	0,285
6 a 8 meses	-0,762	0,188	-0,75	0,541	2,00	<0,001
9 a 11 meses	-0,006	0,991	0,44	0,657	18,00	<0,001
12 a 24 meses	0,812	0,065	2,00	<0,001	19,00	<0,001
Chefe do AF masculino ^†‡^	0,542	0,692	24,00	0.074	3,00	0,041
Prática da agricultura	-2,00	0,170	22,00	<0.001	11,00	<0,001
Reserva alimentar	0,90	0,006	1,00	0,0150	1,00	0,022
Quantidade de gado bovino	-0,07	0,047	-0,03	0,457	-0,02	0,702
Adequação alimentar da criança	-0,17	0,703	-0,23	0,741	-19,00	<0,001
Dificuldade de alimentação no AF ^‡^	0,49	0,113	0,94	0,032	0,16	0,779
Modelo 2 (crianças de 12 a 24 meses, n=125)						
Número de membros no AF ^‡^	-0,92	0,033	-10,68	0,030	-0,43	0,397
Prática da agricultura	28,32	<0,001	26,31	<0.001	10,00	<0,001
Total de produtos para consumo	0,48	0,442	0,62	0,021	0,58	0,054
Reserva alimentar	1,43	0,014	2,09	0,001	1,90	0,007
Quantidade de suínos	29,00	<0,001	-9,08	<0,001	28,91	<0,001
Dificuldade de alimentação no AF ^‡^	1,44	0,010	2,20	<0,001	0,526	0,489

*Estim. = Estimativa; ^†^Aquele que respondeu ao questionário destinado ao chefe do AF; ^‡^AF = Agregado familiar

## Discussão

Neste estudo, foi observado que, em uma região de Moçambique cuja economia é baseada no comércio interno de alimentos, em que a persistência da desnutrição infantil vinha chamando a atenção das autoridades locais com foco na cultura alimentar, a desnutrição se associou não só às variáveis da prática alimentar mas também da disponibilidade local e do acesso aos alimentos. Foram evidenciadas práticas inadequadas de alimentação das crianças, no entanto o papel protetor do aleitamento materno no primeiro ano de vida teve bom desempenho. Foi demostrado que a existência de reserva alimentar era mais importante que a existência de produtos para a venda e também foi evidenciado que, nos agregados familiares chefiados por homens, as crianças estavam menos expostas à desnutrição. O dado que mais chamou a atenção foi que menos de um terço das crianças avaliadas estava livre de qualquer tipo dos agravos nutricionais avaliados e que esses agravos estavam associados a todas as dimensões da SAN consideradas na pesquisa, mostrando a complexidade do problema. Analisou-se a alimentação complementar em 80 países^(23)^, a partir de fontes de dados nacionais, e observou-se que a África Ocidental e a Central apresentaram os piores resultados relativos à diversidade, frequência e adequação do consumo alimentar das crianças e que apenas o aleitamento materno se apresentou com melhores índices nesses locais.

Discutir as práticas alimentares inadequadas é tarefa complicada, começando por destacar que essas tradições alimentares, passadas de mãe para filha, não deixam de ser mecanismos de resiliência diante de um ambiente alimentar desfavorável que vem se perpetuando no continente africano^(1)^. Em estudo anterior, nessa mesma região de Moçambique, foi identificado que a prática do aleitamento materno foi mais frequentemente ensinada de mãe para filha do que pelos profissionais de saúde^(24)^. Em outro estudo no distrito de Sussundenga, foi observado o quanto o patriarcado tem sido prejudicial às mulheres que viviam sobrecarregadas com as tarefas de cuidar dos filhos e das *muchambas* (pequenas roças) e com baixo ou quase nenhum poder de participação nos empreendimentos locais^(25)^. No presente estudo, 12% dos domicílios eram chefiados por mulheres, e o fato de o chefe do AF ser um homem e possuir mais idade que os outros foi fator protetor à desnutrição. Essa característica patriarcal persiste na África desde os tempos pré-coloniais^(26)^.

O aleitamento materno exclusivo em crianças menores de seis meses foi de 67%, um valor expressivo quando se considera que apenas 40% das crianças do mundo são alimentadas exclusivamente no seio até os seis meses de idade^(27)^, embora o ideal fosse 80%. Chamou a atenção ainda que quase todas (97%) estavam em aleitamento continuado até um ano. Estudos apontam que a baixa cobertura do aleitamento materno exclusivo está relacionada a técnicas inadequadas e à carência de atividades integradas de comunicação e educação nutricional^(28-29)^. No presente estudo, importa muito a valorização desse conhecimento tradicional, haja vista o papel das mulheres mais velhas e mais experientes na perpetuação dessa prática.

Claramente a inadequação alimentar da criança se mostrou associada à desnutrição aguda grave e moderada e à desnutrição crônica grave (Tabelas 4 e 5). Quando se buscou associação com a idade e a desnutrição crônica, verificou-se associação negativa dentro das faixas etárias de transição nutricional (6 a 8 meses e 9 a 11 meses) e positiva entre as crianças maiores de um ano (Tabela 5), reforçando a importância do período de transição nutricional que ocorre entre os seis e 11 meses, quando as crianças aos poucos vão se tornando menos dependentes do leite materno. Além disso, é de se notar o comportamento dos dados de adequação do estado nutricional para todos os indicadores, apresentando-se baixos até os cinco meses de idade, mais elevados aos seis e oito meses e voltando aos patamares iniciais nas idades entre nove e 24 meses. Não temos informação do peso ao nascer dessas crianças, que pode ter influenciado a inadequação do estado nutricional nos primeiros meses de vida. É provável que uma maior adequação dos seis aos oito meses tenha sido beneficiada pelo aleitamento exclusivo no início da vida e que, aos poucos, esse ganho tenha sido prejudicado pela inadequação da transição alimentar. Esses achados corroboram as evidências de que a alimentação complementar deve se iniciar aos seis meses, com alimentos variados e frequência adequada, garantindo-se o aleitamento materno até os 24 meses^(30-32)^.

Tradicionalmente, a base da refeição de crianças menos favorecidas vem sendo composta de cereais, porque essa produção e esse acesso têm sido oferecidos pelo ambiente^(3,33-34)^. As papas enriquecidas vêm sendo preconizadas como alternativa^(25)^ e surge então a pergunta: porque as mães insistem em não aderir a essa recomendação de enriquecer as papas? Tal situação merece reflexão ante o fato de que a prática da agricultura é fator de proteção à desnutrição. Os agricultores que conseguiram reservas alimentares tiveram suas crianças protegidas da desnutrição. Embora essas reservas estivessem centradas no milho e feijão, as crianças protegidas contaram, também, com diversidade de animais e verduras para consumo.

Já existem evidências suficientes sobre a importância da diversidade alimentar para a manutenção do bom estado nutricional, em especial a partir do momento em que se realiza a transição alimentar, a qual deve incluir o consumo de frutas e verduras^(35-36)^. Parece aqui que o melhor caminho a seguir é investir no fortalecimento dos agricultores e da agricultura, aprofundando-se o conhecimento das práticas locais e das reais necessidades daqueles que não produzem o suficiente (sementes, mudas, filhotes de animais, outros insumos e tecnologias sociais).

Foi visto que nem todos os AFs comercializavam o que produziam e, mais do que comercializar, produzir o suficiente para ter reserva alimentar permitiu não ter dificuldade com alimentação e, consequentemente, ter crianças bem nutridas. A disponibilidade de variedade de animais para consumo protegeu da desnutrição crônica. Na desnutrição aguda, a criação de gado bovino apareceu negativamente associada à proteção da desnutrição aguda de todas as crianças e a criação de suínos protegeu as crianças maiores de um ano. Além disso, mais que a metade da criação animal nos AF era composta por aves, também utilizadas para consumo. Isso reforça a importância de ter alimentos para consumo, visto que a venda (no caso dos bovinos) não ajudou a proteger da desnutrição. A dificuldade com alimentação na família afetou principalmente as crianças maiores de um ano, no indicador de desnutrição aguda ou no de crônica. Essas crianças maiores estiveram menos protegidas pelo aleitamento materno.

De acordo com o boletim estatístico de pecuária de Moçambique, houve um crescimento da produção de gado bovino em 2022 em cerca de 5% em relação ao ano 2021, incluindo a produção familiar^(37)^. Quanto ao gado bovino, por ser eleito para comercialização, pressupõe-se que as famílias tenham, com a sua venda, a possibilidade de ganhar dinheiro para o autossustento. Parece, no entanto, que, diante do fato de ser a produção dos pequenos animais aquela que se apresenta como fator protetor da desnutrição, o incentivo à pecuária deveria vir no sentido de apoio à produção de pequenos animais como fonte proteica na dieta dos AFs. Contudo, o tema é complexo, pois essas famílias têm necessidades além das alimentares que precisam ser supridas, incluindo também os gêneros alimentícios que não são passíveis de produção local. A venda de alimentos produzidos e de animais (68%) foi identificada como a principal fonte de rendimento dessas famílias - que costumam ser numerosas e, quanto maiores forem, maior será a presença de desnutrição crônica leve e moderada entre crianças maiores de um ano, mais dependentes das reservas de alimento da família. Foi observado que 65% dos AFs apresentavam algum grau de insegurança alimentar.

No que diz respeito às políticas públicas, o governo moçambicano acertadamente priorizou o aleitamento materno exclusivo na Estratégia Nacional de Alimentação Infantil em 2019^(38-39)^. Além disso, com vistas a responder aos objetivos 1 e 2 do Desenvolvimento Sustentável, inspirado na Estratégia de Segurança Alimentar e Nutricional da Comunidade dos Países de Língua Portuguesa (ESAN- CPLP)^(40)^, ele vem seguindo as orientações do Conselho de Segurança Alimentar e Nutricional da Comunidade de Países de Língua Portuguesa (CONSAN- -CPLP)^(41)^ e as diretrizes voluntárias da FAO^(42)^, buscando a erradicação da fome e a promoção da agricultura sustentável, reforçando as políticas públicas já existentes na perspectiva do direito humano à alimentação adequada e sustentabilidade. Para tanto, formulou e implementou vários planos de ação, com maior destaque para o Plano Estratégico para Desenvolvimento do Setor Agrário^(43)^. Por meio do pilar produção, produtividade e competitividade agrária, estabeleceu ações que visam garantir a SAN apoiada na promoção da consistência e acessibilidade de alimentos; investimentos na expansão da reserva física alimentar; estabelecimento e/ou fortalecimento e operacionalização de programas de proteção social que assegurem acesso aos bancos de alimentos pelas comunidades vulneráveis; fortalecimento da implementação e/ou estabelecimento de programas estratégicos de fortificação alimentar, incluindo biofortificação; promoção de mudança social e de comportamento, tendo em vista a diversificação e o balanceamento da dieta e o fortalecimento do monitoramento e a avaliação regular da SAN. Isso mostra a preocupação do governo moçambicano com a SAN, no entanto a implementação dessas políticas ainda não se viu refletida nos AFs da localidade de Mouha e suas crianças. Aqui caberia uma discussão das causas estruturais e globais que afetam o continente africano, das relações diplomáticas de cooperação, entre outras iniquidades historicamente perpetuadas^(44)^.

Considerando a complexidade da determinação da desnutrição infantil, podemos apontar como limitação deste estudo o fato de não ter considerado outros determinantes de saúde, como as condições da moradia, o saneamento, o acesso aos serviços e o cuidado protetivo. Teve-se o cuidado de selecionar crianças a partir das unidades de saúde, sem qualquer diagnóstico de doença associada à desnutrição, e considerou-se que as oportunidades de acesso aos serviços foram as mesmas para toda a população, o que confere validade aos resultados apresentados.

Este estudo aponta um caminho a ser explorado para a compreensão das dinâmicas locais e o fortalecimento desses agricultores e, principalmente, agricultoras, para que obtenham sustento para seus AFs e possam cuidar adequadamente de suas crianças e; mostra, ainda, a necessidade de avanço de conhecimento que contribuirá com políticas públicas mais efetivas.

## Conclusão

A desnutrição de crianças com menos de 24 meses, seja aguda ou crônica, associa-se igualmente à adequação das práticas alimentares, disponibilidade de alimentos no ambiente local e ao seu acesso para o consumo.

## Data Availability

Todos os dados gerados ou analisados durante este estudo estão incluídos neste artigo publicado.

# Translation: Disponibilidad, acceso y prácticas alimentarias asociadas a la desnutrición en niños menores de 24 meses en Mozambique*

**DOI:** 10.1590/1518-8345.7366.4601

**Authors:** Florência Custódio Mondlane, Lara Morena Cardeal, Rogério António de Oliveira, Maria Rita Marques de Oliveira

**Affiliations:** 1 Universidade Estadual Paulista, Faculdade de Medicina, Botucatu, SP, Brasil.; 2 Becaria de la Coordenação de Aperfeiçoamento de Pessoal de Nível Superior (CAPES), Brasil.; 3 Universidade Estadual Paulista, Instituto de Biociências, Botucatu, SP, Brasil.; 4 Becaria del Conselho Nacional de Desenvolvimento Científico e Tecnológico (CNPq), Brasil.

**Keywords:** Nutrición del Niño, Desnutrición, Lactancia Materna, Seguridad Alimentaria, Producción de Alimentos, Práticas Alimentarias

## Abstract

analizar la asociación de la desnutrición con la disponibilidad, el acceso y las prácticas alimentarias de niños menores de 24 meses en Mouha, Mozambique.

estudio observacional de tipo transversal analítico, con una muestra de 284 niños menores de 24 meses cuyos jefes de familia y madres o responsables de la alimentación del niño respondieron, en el domicilio, a formularios estructurados. Se utilizaron modelos *log*-lineales multinomiales para verificar la asociación de las variables predictoras con indicadores antropométricos de desnutrición.

se observó que menos de un tercio de los niños estaba libre de desnutrición. Solo el 23% de los niños presentaron adecuación alimentaria que cumplía con todos los criterios para su grupo etario, la cual se asoció negativamente con la desnutrición aguda y crónica grave; el 67% de los niños recibían lactancia materna exclusiva, y la prevalencia de lactancia continuada se situaba entre el 88% y el 95%. La existencia de reserva alimentaria fue un factor protector contra la desnutrición crónica en todos los grados, confirmada por la práctica de la agricultura y la diversidad de animales criados. El problema alimentario se asoció con la desnutrición aguda y crónica en los niños mayores de un año.

la desnutrición en niños menores de 24 meses se asocia con la adecuación alimentaria, con la disponibilidad de alimentos para el consumo y con el acceso a ellos.

## Destacados

**(1)** Los agregados familiares están compuestos predominantemente por agricultores. **(2)** La desnutrición es el resultado de prácticas alimentarias inadecuadas. **(3)** Niños de más de un año estaban menos protegidos de la desnutrición. **(4)** Menos de un tercio de los niños estaba libre de la desnutrición.

## Introducción

El derecho humano a una alimentación adecuada consta en las principales pautas internacionales y representa uno de los objetivos de desarrollo sostenible más desafiantes, guardando interdependencia con todos los demás. El hambre, la desnutrición y otras formas de mala nutrición han afectado a más de la mitad de la población mundial. En 2022, el hambre afectaba entre 691 y 783 millones de personas en el mundo, según estimaciones de las Naciones Unidas. Aunque se han logrado algunos progresos en otras regiones afectadas, el hambre continuó aumentando en todas las regiones de África, afectando principalmente a los habitantes de las áreas rurales y a los hogares bajo la responsabilidad de mujeres^(1)^.

En todo el mundo, más del 22,3% de los niños presentaban déficit de crecimiento en 2022, siendo que el 6,8% presentaban delgadez y el 5,6% estaban por encima del peso esperado. En Mozambique, eran 2 millones de niños con déficit de crecimiento, lo que representaba aproximadamente el 5,3% de su población total^(1)^. Se estimó que la prevalencia de la desnutrición crónica en edad preescolar en el país era del 37%^(2)^. Este cuadro ha venido siendo agravado por el acceso inadecuado al agua y al saneamiento, principalmente en la región norte del país^(2)^. Una buena nutrición en los primeros años de vida es primordial para el desarrollo y crecimiento pleno de los niños, con el fin de garantizar una vida plena y saludable hasta la edad adulta^(3)^.

Las medidas antropométricas y sus índices derivados son usados como indicadores del estado nutricional, son de fácil obtención y sensibles a las alteraciones de la composición corporal, ampliamente utilizados para evaluar y monitorear la salud nutricional de la población^(4-5)^. La insuficiencia proteico-calórica reciente se asocia a la delgadez y al bajo peso, y el emaciado puede significar desnutrición aguda; por otro lado, la carencia constante de estos nutrientes durante largos períodos resulta en el compromiso del desarrollo infantil, denunciado por la baja estatura o desnutrición crónica^(6-7)^.

La desnutrición materno-infantil presenta consecuencias a corto y largo plazo. La morbimortalidad y el compromiso del desarrollo físico y psicosocial se presentan como consecuencia a corto plazo, resultando en el aumento de la mortalidad infantil y la susceptibilidad a las enfermedades. A largo plazo, la desnutrición puede resultar en dificultades cognitivas, deterioro en el desempeño reproductivo, incidencia de sobrepeso y enfermedades metabólicas en la vida adulta como consecuencia del hambre en la infancia^(8)^.

La desnutrición ha sido evaluada a partir de causas inmediatas, subyacentes y básicas: la ingesta inadecuada de alimentos y las enfermedades se encuentran entre las causas inmediatas de la desnutrición, mientras que la inseguridad alimentaria y la inadecuación del cuidado y de la atención a la salud se encuentran entre las causas subyacentes. Como causas básicas, se encuentran la falta de acceso a las políticas públicas (de salud, educación, trabajo, agraria) y a las tecnologías, condicionadas al insuficiente capital financiero, humano y a las condiciones físicas y sociales del contexto en el que se vive^(9)^.

La Seguridad Alimentaria y Nutricional (SAN) es un factor preponderante en la garantía del buen estado nutricional. La SAN existe cuando todas las personas, en todo momento, tienen acceso físico, social y económico a alimentos seguros, nutritivos y suficientes que satisfagan sus necesidades dietéticas y preferencias alimentarias para una vida activa y saludable, basada en prácticas alimentarias promotoras de salud, respetando la diversidad cultural, es decir, ambiental, cultural, económica y socialmente sostenibles^(10)^. Esta concepción involucra la dimensión de la disponibilidad de alimentos en el contexto ambiental; el acceso físico y financiero en el contexto familiar; el uso en el contexto biológico, afectivo y cultural; la estabilidad y sostenibilidad de los tres primeros y el poder de acción de derecho del sujeto^(10)^.

Los estudios han revelado que la agropecuaria familiar para el autoconsumo contribuye positivamente a la salud y al sustento de las familias y sus niños, y a la reducción de la inseguridad alimentaria^(11)^. Sin embargo, ante la complejidad del proceso de determinación social de la desnutrición, surge la necesidad de profundizar su entendimiento, teniendo en cuenta cuestiones culturales, sociales y ambientales, ya sea de los medios de subsistencia o de las prácticas alimentarias^(12)^.

En el caso de los niños, el papel de la madre o cuidador es muy relevante. Se observó, por ejemplo, que la lactancia materna exclusiva entre niños de 0 a 5 meses y la adecuada transición nutricional eran más perjudicadas en niños cuyas madres trabajaban fuera del hogar^(13)^, pero no es solo eso, se destaca también que, en Mozambique, las dietas son, en su mayoría, a base de cereales, lo que ha limitado la diversidad alimentaria de niños de 6 a 24 meses^(14)^.

Mouha es una localidad del distrito de Sussundenga, en la provincia de Manica en Mozambique, una región que fue muy afectada por eventos climáticos, pero donde se produce la mayor cantidad de alimentos en el país; sin embargo, también es una de las regiones más afectadas por la desnutrición infantil^(15)^. Se postuló que la desnutrición infantil en la región puede deberse a prácticas alimentarias inadecuadas ejercidas por las madres o responsables de la alimentación del niño. En la literatura referente a los países africanos de lengua portuguesa, la información existente sobre la desnutrición corresponde a datos de estudios transversales que poco profundizan en las causas de la desnutrición^(16)^; aún se desconoce si los niños afectados por la desnutrición están sometidos a baja disponibilidad de alimento y poco acceso a él o si las prácticas alimentarias son inadecuadas. Ante la complejidad del proceso de determinación de la desnutrición y la falta de datos en la región, en esta investigación se partió de la hipótesis de que variables relacionadas con la determinación del estado nutricional relativas a la disponibilidad, el acceso y el consumo podrían estar produciendo la desnutrición infantil en Mouha, como probablemente en otras localidades con características similares. Así, el objetivo de esta investigación fue analizar la asociación de la desnutrición con la disponibilidad, el acceso y las prácticas alimentarias de niños menores de 24 meses en Mouha, Mozambique.

## Método

### Tipo de estudio

Se trata de un estudio observacional de tipo transversal analítico que involucró la recolección de información mediante encuesta domiciliaria, siguiendo para la redacción de este documento el *checklist* STROBE (*Strengthening the Reporting of Observational Studies in Epidemiology*).

### Población y lugar de estudio

La población estuvo constituida por niños menores de 24 meses, el jefe del hogar y la madre o el responsable de la alimentación del niño, residentes en la localidad de Mouha, distrito de Sussundenga, provincia de Manica en Mozambique, fueran ellos practicantes o no de la agricultura y ganadería. La economía del distrito se basa en la agricultura, y la localidad está compuesta por 14 barrios, una unidad sanitaria de tipo II (unidad sanitaria pequeña que brinda cuidados de salud primarios en medio rural) y cuatro escuelas primarias.

### Criterio de selección

Fueron invitados los jefes de hogar, las madres o responsables de la alimentación de niños menores de 24 meses registrados en la unidad sanitaria (US), independientemente del tiempo de residencia en la localidad, que no presentaran problemas de salud que afectaran directamente el estado nutricional (HIV/SIDA, tuberculosis, retraso en el desarrollo psicomotor), según lo indicado en la tarjeta de salud del niño o ficha prenatal. Se excluyó del estudio a todos los Agregados Familiares (AF) (esta es una designación específica, con legislación propia, para un agrupamiento familiar en Mozambique, no usual en Brasil) que no completaron los datos de la investigación y/o a niños diagnosticados, *aposteriori*, con problemas que afectaran directamente el estado nutricional.

### Muestra y muestreo

Se empleó un plan de muestreo aleatorio simple por conglomerados, con un poder del 80% y un nivel de significancia del 5%, considerando 22 variables independientes a ser analizadas por regresión lineal múltiple^(17)^, lo que resultó en 234 niños en sus respectivos AF, siendo solo un niño por cada AF. Se consideró un 20% de pérdida (n=340), siendo que, al final, se incluyeron en la investigación 284 AF provenientes de 10 de los 14 barrios existentes. Primero, se contactó a los líderes y/o secretarios para, a continuación, y con la debida autorización, invitar a las familias con niños menores de 24 meses a participar en la investigación.

### Instrumentos utilizados y variables del estudio

Se utilizaron dos formularios estructurados para la recolección de datos, uno aplicado al jefe del AF o su representante y el otro a la madre o responsable de la alimentación del niño. El formulario dirigido al jefe del AF se componía de las secciones: A - con cuestiones relativas a datos demográficos del jefe del AF; sección B - con cuestiones relativas a la producción de cultivos y ganadería; sección C - con preguntas sobre las formas de vida; sección D - con cuestiones de consumo alimentario, incluyendo el recordatorio de 24 h y el recordatorio de una semana; sección E - con cuestiones sobre la evaluación de la inseguridad alimentaria en la que se aplicó la FIES - *Food Insecurity Experience Scale*, desarrollada por la *Food and Agriculture Organization* (FAO) como herramienta global, incluida la validación para Mozambique^(18)^.

El segundo formulario fue dirigido a la madre o responsable de la alimentación del niño, compuesto por las secciones A y B; en la sección A, las cuestiones eran sobre datos demográficos y antropométricos de los niños y, en la sección B, cuestiones sobre lactancia materna exclusiva, lactancia continuada y alimentación complementaria en formato de recordatorio de 24 h y de una semana de los grupos de alimentos consumidos.

Ambos formularios se obtuvieron a partir del instrumento de recolección de datos del Secretariado Técnico de Seguridad Alimentaria y Nutricional (versión 21/02/2018)^(19)^, compuestos de respuestas predefinidas, tales como: sí, no, no sabe, respuestas cortas y respuestas de opción múltiple correspondientes a la realidad del participante.

Los alimentos se agruparon de acuerdo con la clasificación de alimentos de la FAO, según sigue: cereales; vegetales y tubérculos ricos en vitamina A; vegetales y hojas de color verde oscuro; otros vegetales; leguminosas; frutas de color anaranjado o amarillentas; otras frutas; carnes (blancas y rojas); huevos; leche y productos lácteos; pescados y mariscos; vísceras; semillas; aceites y grasas; dulces y azúcar; condimentos y especias^(20)^.

### Variables del estudio

Las variables del estudio se organizaron en cuatro dimensiones:

Variables demográficas: 1) Edad del jefe del AF; 2) Sexo del jefe del AF,; 3) Nivel de escolaridad del jefe del AF; 4) Número de miembros en el AF; 5) Sexo del niño; 6) Número de niños menores de 24 meses en cada AF y 7) Edad del niño.

Estado nutricional: 8) Peso/Edad; 9) Peso/Estatura; 10) Estatura/Edad y 11) Presencia de edema bilateral (ningún caso constatado).

Disponibilidad alimentaria: 12) Práctica o no de la agricultura; 13) Variedad de cultivos, considerando verduras (col, lechuga, amaranto, hoja de calabaza, otro); legumbres (zanahoria, tomate, pimiento, quingombó, repollo, otro); leguminosas (frijoles, maní, otro); cereales (maíz, sorgo, trigo, arroz, otro); oleaginosas (girasol, sésamo, coco, otro); 14) Cultivos para la venta: verduras (col, lechuga, amaranto, hoja de calabaza, otro); legumbres (zanahoria, tomate, pimiento, quingombó, repollo, otro); leguminosas (frijoles, maní, otro); cereales (maíz, sorgo, trigo, arroz, otro); oleaginosas (girasol, sésamo, coco, otro); 15) Duración de la reserva; 16) Dificultades de cultivo; 17) Variedad de animales; 18) Animales para la venta; 19) Adecuación alimentaria.

Acceso alimentario: 20) Principal fuente de ingresos: producción, venta de cultivos y animales; trabajo por cuenta propia (procesamiento, salario, pensiones, remesas, comercio, servicios) y asistencia alimentaria/ayuda/ganancia/trabajo eventual; 21) Dificultades alimentarias, número de meses con dificultades alimentarias; 22) Inseguridad alimentaria, prácticas alimentarias; 23) Adecuación a las recomendaciones nacionales; 24) Lactancia materna exclusiva; 25) Lactancia materna continuada; 26) Introducción de alimentos sólidos, semisólidos o blandos; 27) Diversidad alimentaria mínima y 28) Frecuencia mínima de comidas.

Cabe aclarar que se consideró como “sí” para adecuación alimentaria a aquel niño que, conforme a su edad, recibe alimentación adecuada de acuerdo con las recomendaciones de la Organización Mundial de la Salud (OMS), del Fondo de las Naciones Unidas para la Infancia (UNICEF) y de la FAO (adoptadas por el Ministerio de Salud de Mozambique), siendo que de 0 a 5 meses debe recibir lactancia materna exclusiva, y de 6 a 24 meses debe tener lactancia continuada, contar con diversidad alimentaria mínima y un número mínimo de comidas, según la edad.

Para el análisis de la Inseguridad Alimentaria se utilizaron el cuestionario y la plantilla para análisis en *Excel*® proporcionados por la FAO^(21)^, que contemplan instrumentos validados para el portugués de Mozambique y en dos dialectos. Las variables demográficas, del estado nutricional, de la disponibilidad alimentaria y del acceso alimentario se consideraron como variables independientes, y las de desenlace fueron los índices nutricionales. Para efectos de análisis, la edad del jefe del AF; el tiempo de escolaridad del jefe del AF; el número de miembros en el AF; la edad del niño; el número de niños menores de 24 meses en el AF, y el total de meses con dificultades alimentarias fueron estratificados por categorías.

### Recolección de datos y período

La recolección fue precedida por un estudio piloto para evaluar la percepción de las cuestiones y la interpretación de los datos, así como por una breve presentación de la investigadora al jefe de la localidad y a los jefes de los barrios. La recolección comenzó el 16 de enero de 2023, partiendo del barrio más cercano a la sede de la localidad hacia el más distante y desde el domicilio más alejado hacia el más próximo, con excepción de uno de los barrios más cercanos a la sede, que fue uno de los últimos en ser visitado por falta de medios de comunicación con la dirección local. Primeramente, el líder del barrio informaba a las familias sobre la investigación que se desarrollaría y las fechas previstas para la recolección de datos en cada barrio, y, a continuación, la investigadora se dirigía al domicilio de cada AF, acompañada o no del líder del barrio. La entrevista con cada jefe o representante de los agregados familiares tuvo una duración promedio de 20 minutos, y de 40 minutos con las madres o responsables de la alimentación de los niños, incluyendo la evaluación antropométrica.

### Cuestiones éticas

La investigación comenzó tras la aprobación del Comité Nacional de Bioética para la Salud del Ministerio de Salud de Mozambique, en la Ref. 882/CNBS/22, y la obtención del Consentimiento Informado para participantes mayores y del asentimiento para las madres o responsables de la alimentación de los niños que fueran menores de 18 años. Toda la información, una vez transcrita, analizada e interpretada, fue destruida, destacando que los datos fueron anonimizados para el análisis.

### Análisis de los datos

Los datos recolectados se organizaron en una hoja de cálculo electrónica. Posteriormente, con el fin de identificar patrones y comprender la naturaleza de los datos, se realizaron análisis descriptivos utilizando el lenguaje de programación estadística R, paquete *dplyr* ^(22)^. Las proporciones obtenidas como resultados para las variables cualitativas y cuantitativas se organizaron en tablas, siendo que las variables demográficas, de disponibilidad alimentaria (con excepción de la variable duración de la reserva, que se expresó en promedio) y de acceso alimentario se categorizaron y expresaron en porcentaje.

Para investigar la asociación entre las variables de desenlace y las predictoras, los datos se dividieron para la realización de los análisis. El primer análisis se realizó ajustando modelos *log*-lineales multinomiales, considerando los datos de todos los niños en meses. De forma similar, el segundo ajuste del modelo *log*-lineal multinomial se realizó, considerando los datos que corresponden a la categoría de edad del niño con el filtro de 12 a 24 meses. En este contexto, se consideró cada índice nutricional como variable de desenlace, con el objetivo de determinar cuáles variables predictoras tuvieron mayor impacto en las variables de desenlace, considerando un 5% de significancia.

## Resultados

De las 284 familias estudiadas, la mayoría de los jefes de los AF tenían edad ≥ 41 años (34%). Del total, el 88% eran del sexo masculino, el 51% tenía siete años de estudios y el 4% no había asistido a la escuela; más del 50% de los AF estaban compuestos por seis o más miembros y, en la mayoría (96%), existía solo un niño menor de 24 meses. Del total de niños, el 51% era del sexo masculino y la mayor parte (44%) tenía entre 6 y 24 meses de edad (Tabla 1).

**Tabla 1-:** Datos demográficos de las familias (n = 284). Mouha, MN, Mozambique, 2023

**Variables**	**Categorías**	**Total**	**Porcentaje**
Edad del jefe del AF* (en años)	≤ 20	3	1%
	21-25	55	19%
	26-30	55	19%
	31-35	41	14%
	36-40	34	12%
	≥ 41	96	35%
Sexo del jefe del AF*	Masculino	250	88%
	Femenino	34	12%
Nivel educativo del jefe del AF* (en años)	Sin estudio	12	4%
	Hasta 7 años de estudios	127	45%
	Más de 7 años de estudios	145	51%
Número de miembros en el AF*	≤ 5	132	47%
	6 a 10	130	46%
	≥ 11	22	7%
Sexo del niño	Masculino	144	51%
	Femenino	140	49%
Niños menores de 24 meses en cada AF*	1	273	96%
	≥ 2	11	4%
Edad del niño (en meses)	0 a 5	80	29%
	6 a 8	41	14%
	9 a 11	38	13%
	12 a 24	125	44%

*AF = Agregado familiar

De los 284 niños evaluados, el 51% se clasificaron con una talla adecuada para la edad, el 60% con el peso adecuado para la talla y el 63% con el peso adecuado para la edad. Sin embargo, al considerar el conjunto de indicadores con los niños distribuidos por rango de edad, la proporción de niños con todos los índices adecuados fue inferior, variando del 42% entre los de 6 a 8 meses al 21% entre los de 12 a 24 meses, según la Tabla 2.

**Tabla 2-:** Estado nutricional y alimentario de los niños menores de 24 meses (n = 284). Mouha, MN, Mozambique, 2023

**Clasificación del estado nutricional de los niños**										
Talla/Edad			Peso/Talla				Peso/Edad			
A*	L ^†^	M+G ^‡^	A	E ^§^	L ^†^	M+G ^‡^	A*	E ^§^	L ^†^	M+G ^‡^
156	75	53	176	76	23	9	180	42	53	9
55%	26%	19%	62%	27%	8%	3%	63%	15%	19%	3%
Adecuación del estado nutricional en todos los índices										
0 a 5 meses (n=80)			9 a 11 meses (n=38)				12 a 24 meses (n=125)			
21 (26%)			9 (24%)				26 (21%)			
**Prácticas alimentarias**										
Adecuación alimentaria de acuerdo con las recomendaciones nacionales										
0 a 5 meses (n=80)			9 a 11 meses (n=38)				12 a 24 meses (n=125)			
53 (67%)			4 (11%)				7 (6%)			
Lactancia materna exclusiva										
0 a 5 meses (n=80)			9 a 11 meses (n=38)				12 a 24 meses (n=125)			
53 (67%)			-				-			
Lactancia materna continuada										
0 a 5 meses (n=80)			9 a 11 meses (n=38)				12 a 24 meses (n=125)			
-			36 (95%)				110 (88%)			
Introducción de alimentos sólidos, semisólidos o blandos										
0 a 5 meses (n=80)			9 a 11 meses (n=38)				12 a 24 meses (n=125)			
-			-				-			
Diversidad alimentaria mínima										
0 a 5 meses (n=80)			9 a 11 meses (n=38)				12 a 24 meses (n=125)			
-			12 (30%)				30 (24%)			
Frecuencia mínima de comidas										
0 a 5 meses (n=80)			9 a 11 meses (n=38)				12 a 24 meses (n=125)			
-			17 (45%)				74 (59%)			

*A = Adecuado; ^†^L = Leve; ^‡^M+G = Moderado + Grave; ^§^E = Exceso

En cuanto a la práctica alimentaria, al analizar la adecuación según lo preconizado por las directrices adoptadas en el país para cada rango etario, solo 66 (23%) de los niños cumplían con todos los criterios. Entre los niños de 0 a 5 meses, el 67% recibían lactancia materna exclusiva; entre los de 6 a 8 meses, el 88% estaban en lactancia continuada, el 56% tenían la frecuencia mínima de comidas y el 15% presentaban diversidad alimentaria mínima. En el rango etario de 9 a 11 meses, el 95% estaban en lactancia continuada, el 30% y el 45% tenían diversidad alimentaria mínima y frecuencia mínima de comidas, respectivamente. De los 125 niños de 12 a 24 meses, el 88% estaban en lactancia continuada, el 24% tenían diversidad alimentaria mínima y el 59% presentaban frecuencia mínima de comidas (Tabla 2).

El levantamiento de la disponibilidad alimentaria en los AF mostró que la mayoría de las familias practicaba la agricultura, siendo que los cereales y las verduras eran los cultivos más producidos, con 98% y 73%, respectivamente, y las oleaginosas se producían en menor cantidad. De los cultivos producidos, las familias comercializaban más cereales y leguminosas (36,6%). Los AF reservaban cereales y leguminosas para consumo y eventual venta, siendo el maíz, el frijol catarina y el frijol nhemba los cultivos que más reservaban. La duración anual media del maíz era de 1,53,8 meses y la de los frijoles de 2,84,6 meses. Se observó que el 42% de las familias presentaban dificultades para el cultivo de vegetales, siendo la insuficiencia de semillas/mudas la principal dificultad.

En cuanto a la cría de animales, las familias criaban más aves, destacándose las gallinas (87%), el ganado bovino (44%) y, en menor cantidad, los cerdos. De las especies criadas, el ganado bovino y caprino eran los más comercializados.

En la Tabla 3, sobre el acceso al alimento, la producción y venta eran la principal fuente de ingresos de las familias; incluso con la presencia de producción, el 39,1% de las familias presentaban dificultad alimentaria durante el año, faltando comida, en promedio, durante tres meses.

**Tabla 3-:** Disponibilidad de alimento y acceso a él referidos por los jefes del AF (n = 284). Mouha, MN, Mozambique, 2023

**Variables**	**Categorías**	**Total**	**Porcentaje**
**Indicadores de disponibilidad alimentaria**			
Práctica de la agricultura	Sí	281	99%
Reserva alimentaria	Sí	104	37%
Dificultad de cultivo	Sí	119	42%
Dificultad alimentaria	Sí	111	39%
Variedad de cultivo	Verduras	206	72%
	Legumbres	90	32%
	Leguminosas	162	57%
	Cereales	279	98%
	Oleaginosas	71	25%
Cultivos para venta	Leguminosas	122	43%
	Cereales	145	51%
Variedad de animales	Bovino	125	44%
	Porcino	5	2%
	Caprino	109	38%
	Aves	246	87%
Animales para venta	Bovino	125	44%
	Porcino	5	2%
	Caprino	109	38%
	Aves	95	34%
Duración de la reserva	Maíz*	1,5	3,8
	Frijoles*	2,8	4,6
**Indicadores de acceso al alimento**			
Principal fuente de ingresos	Venta de la producción	193	68%
	Trabajo por cuenta propia	84	30%
	Asistencia alimentaria/ayuda/ganancia/trabajo eventual	7	2%
Dificultades con la alimentación en el año	≤3 meses	91	32,0%
	≥4 meses	22	7,7%
Inseguridad alimentaria	Seguridad	99	35%
	Leve	89	31%
	Moderada	35	12%
	Grave	61	22%

*Media y desviación estándar

Del total de AF, solo el 35% presentaba seguridad alimentaria, el 31% inseguridad alimentaria leve, el 12% inseguridad alimentaria moderada y el 22% inseguridad alimentaria grave, según la Tabla 3.

Cuando se analizaron todas las variables potenciales predictoras tomando los indicadores del estado nutricional como desenlace, se presentaron los modelos que mejor se ajustaron (peso/talla o desnutrición aguda y talla/edad o desnutrición crónica), destacándose que los modelos obtenidos para el indicador peso/edad no fueron priorizados, ya que el peso representaba una medida genérica de asociación de los indicadores talla/edad y peso/talla y, por lo tanto, se consideraron menos sensibles. Se construyeron modelos para los niños y para niños de 12 a 24 meses más dependientes de las reservas alimentarias de la familia.

Tener un jefe del AF de mayor edad aparece como factor protector en la desnutrición aguda leve, independientemente de la edad del niño. Entre los niños de 12 a 24 meses, que el jefe del AF sea del sexo masculino aparece como factor protector en la desnutrición aguda leve. El número de miembros del AF no mostró asociación con la desnutrición aguda, pero se asoció negativamente con la desnutrición crónica leve y moderada, independientemente de la edad del niño. Por otro lado, el total de productos para el consumo fue un factor protector en la desnutrición crónica moderada y grave. En la desnutrición aguda moderada y grave de los niños de 12 a 24 meses, la variedad de animales criados, a partir de dos animales, fue un factor protector.

Presentar una mayor cantidad de productos para la venta se asocia negativamente con la desnutrición aguda leve entre todos los niños, y la adecuación alimentaria aparece como factor protector de la desnutrición aguda moderada y grave y de la desnutrición crónica moderada entre todas los niños, según muestra la Tabla 4.

**Tabla 4-:** Modelos *log*-lineales multinomiales tomando el índice peso/talla como variable de desenlace, considerando todos los niños de 12 a 24 meses. Mouha, MN, Mozambique, 2023

Variables	Índice peso/Talla			
	Leve		Moderado+grave	
	Estimación	*p-valor*	Estimación	*p-valor*
Modelo 1 (todos los niños, n=284)				
Edad del jefe del AF*	0,41	0,022	0,23	0,396
Número de miembros en el AF*	-0,36	0,395	0,58	0,273
Total de productos para la venta	-0,37	0,019	0,39	0,100
Adecuación alimentaria del niño	0,71	0,159	1, 60	0,025
Modelo 2 (niños de 12 a 24 meses, n=125)				
Jefe del AF* del sexo masculino	16,00	<0,001	-2,00	0,172
Práctica de la agricultura	17,00	<0,001	18,00	<0,001
Productos para venta	-0,41	0,186	0,37	0,396
Cría de animales	0,58	0,664	4,00	<0,001
Una variedad de animales para consumo	-2,00	0,150	-8,00	<0,001
Dos variedades de animales para consumo	-2.07	0,183	13.19	<0,001
Tres variedades de animales para consumo	-0,89	0,572	14,00	<0,001
Dificultad de alimentación en el AF*	-0,05	0,947	-68,00	<0,001

*AF = Agregado familiar

El hecho de que el niño sea del sexo masculino representó un factor protector en la desnutrición crónica moderada para todos los niños. La edad del niño de 6 a 11 meses está asociada con la desnutrición crónica grave, y la de 12 a 24 meses, con la desnutrición crónica moderada grave, según el modelo 1. La presencia del hombre en casa (jefe del AF masculino) fue factor protector en la desnutrición crónica grave de todos los niños.

La práctica de la agricultura aparece como factor protector en la desnutrición aguda de los niños de 12 a 24 meses y como factor protector de la desnutrición crónica de todos los niños en las clasificaciones moderada y grave y, en el modelo para los niños de 12 a 24 meses, en las clasificaciones leve, moderada y grave de la desnutrición crónica. Tener productos para la venta no mostró asociación con las variables de desenlace en ninguno de los modelos.

La cantidad de ganado bovino, en el modelo 1, se asoció negativamente con la desnutrición crónica leve, mientras que en el modelo 2, la cantidad de cerdos apareció como factor protector en la desnutrición leve, moderada y grave.

La dificultad de alimentación en el AF se asoció negativamente con la desnutrición aguda moderada y grave y con la desnutrición crónica leve y moderada. En la desnutrición crónica, tanto en el modelo 1 como en el modelo 2, la presencia de reserva alimentaria fue factor de protección en todas las clasificaciones de la desnutrición crónica (Tabla 5).

**Tabla 5-:** Modelos *log*-lineales multinomiales tomando el índice talla/edad como variable de desenlace, considerando todos los niños de 12 a 24 meses. Mouha, MN, Mozambique, 2023

Variables	Índice talla/edad					
	Leve		Moderado		Grave	
	Estim.*	*p- valor*	Estim.*	*p- valor*	Estim.*	*p-valor*
Modelo 1 (todos los niños, n=284)						
Sexo masculino	0,587	0,058	11,00	0,016	0,58	0,285
6 a 8 meses	-0,762	0,188	-0,75	0,541	2,00	<0,001
9 a 11 meses	-0,006	0,991	0,44	0,657	18,00	<0,001
12 a 24 meses	0,812	0,065	2,00	<0,001	19,00	<0,001
Jefe del AF masculino ^†‡^	0,542	0,692	24,00	0.074	3,00	0,041
Práctica de la agricultura	-2,00	0,170	22,00	<0.001	11,00	<0,001
Reserva alimentaria	0,90	0,006	1,00	0,0150	1,00	0,022
Cantidad de ganado bovino	-0,07	0,047	-0,03	0,457	-0,02	0,702
Adecuación alimentaria del niño	-0,17	0,703	-0,23	0,741	-19,00	<0,001
Dificultad de alimentación en el AF ^‡^	0,49	0,113	0,94	0,032	0,16	0,779
Modelo 2 (niños de 12 a 24 meses, n=125)						
Número de miembros en el AF ^‡^	-0,92	0,033	-10,68	0,030	-0,43	0,397
Práctica de la agricultura	28,32	<0,001	26,31	<0.001	10,00	<0,001
Total de productos para el consumo	0,48	0,442	0,62	0,021	0,58	0,054
Reserva alimentaria	1,43	0,014	2,09	0,001	1,90	0,007
Cantidad de cerdos	29,00	<0,001	-9,08	<0,001	28,91	<0,001
Dificultad de alimentación en el AF ^‡^	1,44	0,010	2,20	<0,001	0,526	0,489

*Estim. = Estimación; ^†^Aquel que respondió al cuestionario destinado al jefe del AF; ^‡^AF = Agregado familiar

En los ajustes para el indicador peso/edad, las variables predictoras fueron la edad del niño, el sexo del jefe del AF y el número de miembros en el AF para todos, y la práctica de la agricultura y la adecuación alimentaria para niños de 12 a 24 meses (datos no mostrados).

## Discusión

En este estudio se observó que, en una región de Mozambique cuya economía se basa en el comercio interno de alimentos, donde la persistencia de la desnutrición infantil venía llamando la atención de las autoridades locales con foco en la cultura alimentaria, la desnutrición se asoció no solo con las variables de la práctica alimentaria, sino también con la disponibilidad local y el acceso a los alimentos. Se evidenciaron prácticas inadecuadas de alimentación de los niños; sin embargo, el papel protector de la lactancia materna en el primer año de vida tuvo buen desempeño. Se demostró que la existencia de reserva alimentaria era más importante que la existencia de productos para la venta y también se evidenció que, en los agregados familiares encabezados por hombres, los niños estaban menos expuestos a la desnutrición. El dato que más llamó la atención fue que menos de un tercio de los niños evaluados estaba libre de cualquier tipo de agravios nutricionales evaluados y que estos agravios estaban asociados a todas las dimensiones de la SAN consideradas en la investigación, mostrando la complejidad del problema. Se analizó la alimentación complementaria en 80 países^(23)^, a partir de fuentes de datos nacionales, y se observó que África Occidental y Central presentaron los peores resultados relativos a la diversidad, frecuencia y adecuación del consumo alimentario de los niños, y que solo la lactancia materna se presentó con mejores índices en esos lugares.

Discutir las prácticas alimentarias inadecuadas es una tarea complicada, empezando por destacar que esas tradiciones alimentarias, transmitidas de madre a hija, no dejan de ser mecanismos de resiliencia frente a un ambiente alimentario desfavorable que se viene perpetuando en el continente africano^(1)^. En un estudio previo, en esa misma región de Mozambique, se identificó que la práctica de la lactancia materna fue enseñada con mayor frecuencia de madre a hija que por los profesionales de salud^(24)^. En otro estudio en el distrito de Sussundenga, se observó cuánto el patriarcado ha sido perjudicial para las mujeres, quienes vivían sobrecargadas con las tareas de cuidar a los hijos y de las *muchambas* (pequeñas chacras) y con poco o casi ningún poder de participación en los emprendimientos locales^(25)^. En el presente estudio, el 12% de los hogares estaban encabezados por mujeres, y el hecho de que el jefe del AF fuera hombre y tuviera mayor edad que los demás fue factor protector de la desnutrición. Esa característica patriarcal persiste en África desde tiempos precoloniales^(26)^.

La lactancia materna exclusiva en niños menores de seis meses fue del 67%, un valor expresivo si se considera que solo el 40% de los niños en el mundo son alimentados exclusivamente al seno hasta los seis meses de edad^(27)^, aunque lo ideal sería el 80%. Llamó aún la atención que casi todos (97%) estaban en lactancia continuada hasta un año. Estudios señalan que la baja cobertura de la lactancia materna exclusiva está relacionada con técnicas inadecuadas y la carencia de actividades integradas de comunicación y educación nutricional^(28-29)^. En el presente estudio, es muy importante la valoración de ese conocimiento tradicional, dada la función de las mujeres mayores y más experimentadas en la perpetuación de esa práctica.

Claramente, la inadecuación alimentaria del niño se mostró asociada con la desnutrición aguda moderada y grave, y con la desnutrición crónica grave (Tabla 4 y Tabla 5). Cuando se buscó asociación con la edad y la desnutrición crónica, se verificó una asociación negativa dentro de las franjas etarias de transición nutricional (6 a 8 meses y 9 a 11 meses) y positiva entre los niños mayores de un año (Tabla 5), lo que refuerza la importancia del periodo de transición nutricional que ocurre entre los seis y 11 meses, cuando los niños, gradualmente, van siendo menos dependientes de la leche materna. Además, cabe notar el comportamiento de los datos de adecuación del estado nutricional para todos los indicadores, los cuales se mostraron bajos hasta los cinco meses de edad, más elevados entre los seis y ocho meses y regresaron a los niveles iniciales en las edades de nueve a 24 meses. No se cuenta con información sobre el peso al nacer de estos niños, lo cual pudo haber influido en la inadecuación del estado nutricional en los primeros meses de vida. Es probable que la mayor adecuación observada entre los seis y ocho meses haya sido favorecida por la lactancia materna exclusiva al inicio de la vida y que, gradualmente, este beneficio se vio perjudicado por la inadecuación de la transición alimentaria. Estos hallazgos corroboran las evidencias de que la alimentación complementaria debe iniciarse a los seis meses, con alimentos variados y una frecuencia adecuada, garantizando la lactancia materna hasta los 24 meses^(30-32)^.

Tradicionalmente, la base de la alimentación de los niños menos favorecidos ha estado compuesta por cereales, dado que es esta producción y el acceso que el entorno ofrece lo que predomina^(3,33-34)^. Las papillas enriquecidas han sido preconizadas como alternativa^(25)^, lo que plantea la pregunta: ¿por qué las madres insisten en no adherirse a la recomendación de enriquecer las papillas? Tal situación merece reflexión, considerando que la práctica de la agricultura es un factor de protección contra la desnutrición. Los agricultores que consiguieron reservas alimentarias lograron que sus niños estuvieran protegidos de la desnutrición. Aunque estas reservas se centraban en el maíz y el frijol, los niños protegidos también contaron con una diversidad de animales y verduras para consumo.

Ya existen evidencias suficientes sobre la importancia de la diversidad alimentaria para mantener un buen estado nutricional, especialmente a partir del momento en que se inicia la transición alimentaria, la cual debe incluir el consumo de frutas y verduras^(35-36)^. Parece, pues, que el mejor camino a seguir es invertir en el fortalecimiento de los agricultores y de la agricultura, profundizando en el conocimiento de las prácticas locales y en las reales necesidades de aquellos que no producen lo suficiente (semillas, plántulas, crías de animales, otros insumos y tecnologías sociales).

Se observó que no todos los AF comercializaban lo que producían y, más que la venta, producir lo suficiente para tener una reserva alimentaria permitió no tener dificultades con la alimentación y, en consecuencia, contar con niños bien nutridos. Contar con variedad de animales para consumo fue lo que protegió contra la desnutrición crónica. En la desnutrición aguda, la cría de ganado bovino se asoció negativamente con la protección frente a la desnutrición aguda en todos los niños, mientras que la cría de cerdos protegió a los niños mayores de un año. Además, más de la mitad de la cría animal en los AF estaba compuesta por aves, las cuales también se utilizaban para el consumo. Esto refuerza la importancia de disponer de alimentos para consumo, ya que la venta (en el caso de los bovinos) no ayudó a proteger contra la desnutrición. La dificultad con la alimentación en el hogar afectó principalmente a los niños mayores de un año, tanto en el indicador de desnutrición aguda como en el de crónica, dado que estos niños estuvieron menos protegidos por la lactancia materna.

De acuerdo con el boletín estadístico de ganadería de Mozambique, hubo un crecimiento de la producción de ganado bovino en 2022 en aproximadamente un 5% en relación con el año 2021, incluyendo la producción familiar^(37)^. En cuanto al ganado bovino, al ser elegido para la comercialización, se presupone que las familias tienen, con su venta, la posibilidad de ganar dinero para el autosustento. Sin embargo, parece que, dado que es la producción de los animales pequeños la que se presenta como factor protector contra la desnutrición, el incentivo a la ganadería debería orientarse hacia el apoyo a la producción de pequeños animales como fuente proteica en la dieta de los AF. No obstante, el tema es complejo, ya que estas familias tienen necesidades que deben ser cubiertas, además de las alimentarias, incluyendo también aquellos productos alimenticios que no son susceptibles de producción local. La venta de alimentos producidos y animales (68%) fue identificada como la principal fuente de ingreso de estas familias, que suelen ser numerosas y, cuanto mayor es su tamaño, mayor será la presencia de desnutrición crónica leve y moderada entre los niños mayores de un año, más dependientes de las reservas de alimento de la familia. Se observó que el 65% de los AF presentaban algún grado de inseguridad alimentaria.

En lo que respecta a las políticas públicas, acertadamente, el gobierno mozambiqueño priorizó la lactancia materna exclusiva en la Estrategia Nacional de Alimentación Infantil en 2019^(38-39)^. Además, con el propósito de responder a los objetivos 1 y 2 del Desarrollo Sostenible, inspirado en la Estrategia de Seguridad Alimentaria y Nutricional de la Comunidad de Países de Lengua Portuguesa (ESAN-CPLP)^(40)^, ha seguido las orientaciones del Consejo de Seguridad Alimentaria y Nutricional de la Comunidad de Países de Lengua Portuguesa (CONSAN-CPLP)^(41)^ y las directrices voluntarias de la FAO^(42)^, buscando la erradicación del hambre y la promoción de la agricultura sostenible, reforzando las políticas públicas ya existentes en la perspectiva del derecho humano a la alimentación adecuada y la sostenibilidad. Para ello, formuló e implementó varios planes de acción, con mayor destaque para el Plan Estratégico para el Desarrollo del Sector Agrario^(43)^. Por medio del pilar de producción, productividad y competitividad agraria, se establecieron acciones que pretenden garantizar la SAN apoyada en la promoción de la consistencia y accesibilidad de alimentos; inversiones en la expansión de la reserva física alimentaria; el establecimiento y/o fortalecimiento y operativización de programas de protección social que aseguren el acceso a los bancos de alimentos por parte de las comunidades vulnerables; el fortalecimiento de la implementación y/o establecimiento de programas estratégicos de fortificación alimentaria, incluida la biofortificación; la promoción del cambio social y de comportamiento con foco a la diversificación y el balance de la dieta, y el fortalecimiento del monitoreo y la evaluación regular de la SAN. Esto muestra la preocupación del gobierno mozambiqueño con la SAN; sin embargo, la implementación de estas políticas aún no se ha reflejado en los AF de la localidad de Mouha y en sus niños. Aquí cabría una discusión sobre las causas estructurales y globales que afectan al continente africano, de las relaciones diplomáticas de cooperación, entre otras inequidades históricamente perpetuadas^(44)^.

Considerando la complejidad de la determinación de la desnutrición infantil, podemos señalar, como limitación de este estudio, no haber considerado otros determinantes de salud, tales como las condiciones de la vivienda, el saneamiento, el acceso a los servicios y el cuidado protector. Se tuvo el cuidado de seleccionar niños a partir de las unidades de salud, sin ningún diagnóstico de enfermedad asociada a la desnutrición, y se consideró que las oportunidades de acceso a los servicios fueron las mismas para toda la población, lo que confiere validez a los resultados presentados. Este estudio señala un camino a explorar para la comprensión de las dinámicas locales y el fortalecimiento de esos agricultores y, principalmente, agricultoras, para que obtengan sustento para sus AF y puedan cuidar adecuadamente de sus niños, y además, la necesidad de avanzar en el conocimiento que contribuya con políticas públicas más efectivas.

## Conclusión

La desnutrición de niños menores de 24 meses sea aguda o crónica, se asocia de igual forma a la adecuación de las prácticas alimentarias, a la disponibilidad de alimentos en el entorno local y a su acceso para el consumo.

## Data Availability

Todos los datos generados o analizados durante este estudio están incluidos en este artículo publicado.
